# Effect of the suspension of Ag-incorporated TiO_2_ nanoparticles (Ag-TiO_2_ NPs) on certain growth, physiology and phytotoxicity parameters in spinach seedlings

**DOI:** 10.1371/journal.pone.0244511

**Published:** 2020-12-29

**Authors:** Fernando Gordillo-Delgado, Jakeline Zuluaga-Acosta, Gonzalo Restrepo-Guerrero

**Affiliations:** 1 Applied Science Research Group for the Development of the Eco-Region (GICADE) Assigned to Interdisciplinary Institute of Sciences, University of Quindío, Armenia, Quindío, Colombia; 2 Program of Electronic Instrumentation Technology of Faculty of Basic Science and Technology, University of Quindío, Armenia, Colombia; VIT University, INDIA

## Abstract

In this work, the effect of the inoculation of silver-incorporated titanium dioxide nanoparticles (Ag-TiO_2_ NPs) in spinach seeds was evaluated on certain growth, physiology and phytotoxicity parameters of the plants. This is an important crop for human consumption with high nutritional value due to their low calorie and fat content, providing various vitamins and minerals, especially iron. These NPs were obtained by means of the sol-gel method and heat treatment; the resulting powder material was characterized using X-ray diffraction and scanning electron microscopy and the influence of these NPs on plants was measured by estimating the germination rate, monitoring morphological parameters and evaluating phytotoxicity. The photosynthetic activity of the spinach plants was estimated through the quantification of the Ratio of Oxygen Evolution (ROE) by the photoacoustic technique. Samples of TiO_2_ powder with particle size between 9 and 43 nm were used to quantify the germination rate, which served to determine a narrower size range between 7 and 26 nm in the experiments with Ag-TiO_2_ NPs; the presence of Ag in TiO_2_ powder samples was confirmed by energy-dispersive X-ray spectroscopy. The analysis of variance showed that the dependent variable (plant growth) could be affected by the evaluated factors (concentration and size) with significant differences. The statistical trend indicated that the application of the Ag-TiO_2_ NPs suspension of lowest concentration and smallest particle size could be a promoting agent of the growth and development of these plants. The inoculation with NPs of 8.3 nm size and lowest concentration was related to the highest average ROE value, 24.6 ± 0.2%, while the control group was 20.2 ± 0.2%. The positive effect of the Ag-TiO_2_ NPs treatment could be associated to the generation of reactive oxygen species, antimicrobial activity, increased biochemical attributes, enzymatic activity or improvements in water absorption.

## Introduction

The demand for healthy food products with nutritional quality has increased [1]. In particular, spinach is consumed mainly for its nutritional content [2], given that it is a low-calorie food that contains protein and a small amount of fat, and that it provides fiber and micronutrients such as vitamin C, vitamin A and minerals, especially iron [3]. Spinach production worldwide reached 26.25 million tons in 2018, China being the largest producer with 23.79 million tons [4]. The yield of these conventional crops is frequently based on the use of agrochemical compounds that have adverse effects on the environment [5]; consequently, the search for effective and less polluting alternatives is necessary to mitigate the impact of production. On that subject, the areas of biotechnology and nanotechnology have contributed greatly in recent years [6].

Titanium dioxide (TiO_2_) is one of the most abundant compounds on our planet [7] and it is widely used as a photocatalyst for the conversion of solar energy and for environmental applications due to its characteristics of chemical stability, high refractive index, low cost and lack of toxicity [8]. The potential of this material to promote plant growth and germination, to increase the resistance to stress and to facilitate the absorption of water and oxygen in the seeds has been evaluated recently [9].

Nanoparticles (NPs) are materials that range from 1 to 100 nm in size; to this scale the surface area to volume ratio increases drastically, which enhances the reaction efficiency in applications related to interactions with external substances, such as photocatalysis [10].

An effective strategy to improve the performance of TiO_2_ NPs in photocatalytic applications is based on the shifting of their band-gap energy to the visible region of the spectrum, through the incorporation of impurities and the increase of the surface/volume ratio [11]. Silver (Ag) is one of these possible dopants, which is used in an isolated way for its microbicide and catalytic activity [12]; in particular, the NPs of this material can be used in crops to attack diseases caused by pathogenic microorganisms [13]; these nanostructures generate oxidative stress in bacteria due to the disruption of the electronic transport chain, which is a consequence of high affinity with the cell membrane [14]. On the other hand, doping TiO_2_ with Ag prevents the fast recombination process of electron-hole pairs, which improves its photoactivity [13, 15].

Although nanotechnology has great potential for the development of plant science and production systems [9], the exposure to NPs can have positive or negative effects, depending on factors such as plant species and dosage, as well as particle size and shape of the NPs [16–18]. For example, a growth promoting effect on chili plants, applying doses of Cu NPs at concentrations below 50 ppm, through foliar spray and root absorption was found [19], while other authors [20] observed an opposite reaction when they used 1000 ppm of CuO and ZnO NPs on cucumber seedlings (*Cumumis sativus*), which led to a reduction of plant biomass of 75 and 35% regarding control, respectively. In this case, they found a positive correlation between the bioavailability and potential of oxidative stress in plants, and the particle size, concentration and species of NPs; in addition, the same authors found that the bioaccumulation in plant tissues and the activity of superoxide dismutase and peroxidase in root cells were proportional to the concentration of the nanosized material [20]. In others works, several authors have found that the excess of metal and metal oxide NPs is harmful to plants, but at low doses they can be beneficial for plants. For instance, the ZnO NPs treatment at 1000 ppm concentration had a positive effect on the seedlings by promoting seed germination and the vigor of the seedlings, as well as increasing the chlorophyll content in the leaves; but at 2000 ppm concentration the NPs had a negative and toxic effect on the growth and yield of peanut [21].

The objective of this work was to give an initial approach to the study of the interaction between nanometric titanium dioxide with silver incorporation and spinach seedlings, studying some growth, physiology and phytotoxicity parameters.

## Materials and methods

### Synthesis and characterization of NPs

The Ag-TiO_2_ NPs were obtained using the sol-gel method [22]. In the synthesis of this powder material, 33 mL of methanol (Merck), 5 mL of titanium tetraisopropoxide (Sigma-Aldrich 97%) and 0.04 g of silver nitrate (Sigma-Aldrich 99%) were mixed under constant stirring until complete dissolution; then, 2 mL of distilled water were added dropwise and stirring continued for 5 minutes. This mixture was allowed to dry at room temperature for a week and the obtained solid was ground with mortar and pestle and exposed to heat treatment at temperatures of 300, 400, 500 and 600°C for two hours in order to dehydrate the Ti(OH)_4_, obtained from alkoxide hydrolysis, and to grow anatase TiO_2_ crystal structure [23]. TiO_2_ NPs were synthesized through the same method, using titanium tetraisopropoxide (MERCK 98%) as a precursor; then, the heat treatment was carried out at temperatures of 400, 500, 600 and 700°C for two hours. The samples were characterized by X-ray diffraction (XDR) and the average particle sizes were estimated using Scherrer´s equation [24]. The composition of Ag-TiO_2_ samples was analyzed with Scanning Electron Microscopy-Energy Dispersive X-Ray Spectroscopy (SEM-EDS).

### Seed inoculation and morphological analysis of plants

**Fig 1:**
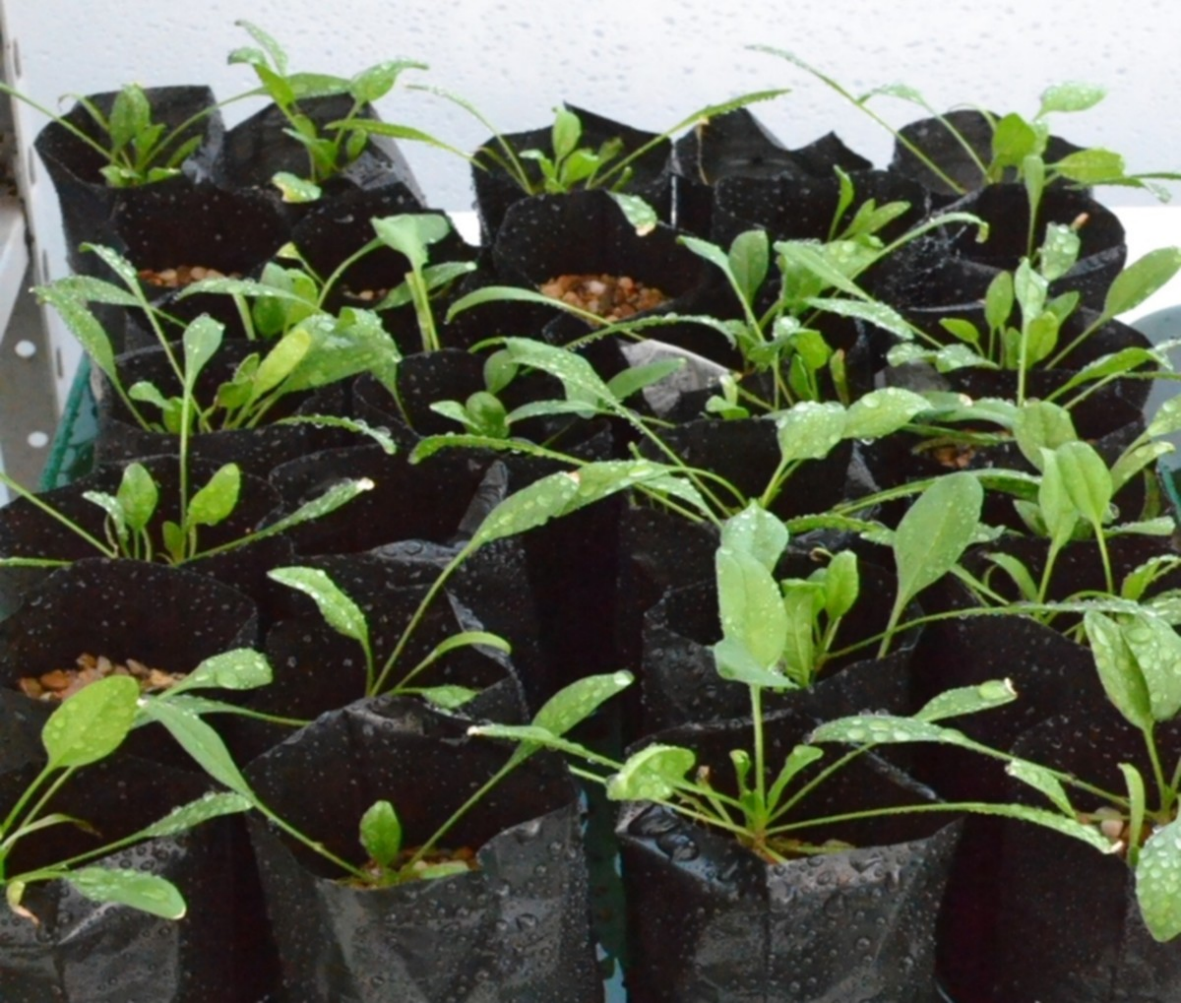
Image of the spinach plants treated with the Ag-TiO_2_ NPs suspension of 7.3 nm and 0.25% concentration, under greenhouse condition.

The suspensions of Ag-TiO_2_ NPs in distilled water at concentrations 2500, 20000, 40000 and 60000 mg/L were treated in an ultrasound bath for a period of three hours; the spinach seeds (S. oleracea) of the Viroflay variety were submerged into these suspensions for three days under daylight exposure for alternate periods of two hours. After this process, the seeds were kept on absorbent paper with daily watering until germination. Then, 10 plants per treatment were grown during 16 days in plastic bags which were pierced to avoid water stagnation, and which contained a previously sterilized hydroponic substrate. The seedlings grew under greenhouse conditions with 64.0 ± 0.1% relative humidity and 24°C; 60 mL per day of the fertilizer and water mixture were added to each plant of each treatment, including those of the control group. In Fig 1, an image of these plants is shown.

A conventional synthetic fertilizer was used: a mixture of 0.37 g urea and 0.31 g nitrogen-phosphorus-potassium (NPK) diluted in 100 mL of distilled water was applied to each plant daily; the equivalence by surface area and percentage for primary macronutrients, to produce 1000 kg/ha is 20 kg/ha of NPK (8-24-16) and 10 kg/ha of urea. Subsequently, morphological measurements were taken daily for sixteen days, recording the number, length and width of the leaves produced and the height of the plant. The results were validated using a multi-factorial analysis of variance with a significance level of 95%; the factors were particle size (7.3, 8.3, 10.9 and 26 nm), concentration (0.25, 2, 4 and 6%) and growth parameters (plant height, width and length of leaves and number of emitted leaves); the number of applied treatments was 64, not counting the control group, each one with 10 repetitions. The used post hoc analysis was the Tukey's test for mean comparison.

### Titanium dioxide nanoparticles phytotoxicity assessment

In a first experiment, the suspensions of TiO_2_ without the incorporation of silver were used at concentrations of 0.25, 2, 4 and 6%; for each treatment, 20 spinach seeds were arranged in order to define the appropriate range of this parameter in a following experiment that allowed to study the effect of silver incorporated NPs on the plant growth. The inoculation process was done as described in the previous section; all seeds were supplied daily with distilled water. The germination was considered achieved when the root was 2 mm long and the corresponding parameters were calculated as reported by Feizi *et al*. [25]. The Average Germination Time (AGT) was calculated using the equation, *AGT* = ∑*FX*/∑*F*, where *F* is the number of seeds germinated at time *X*, which is given in days since inoculation. The Germination Rate (GR) was found using the equation GR = (a/1) + (b-a/2) + (c-b/3) + … + (n-(n-1/N)), where a, b, c, …, n is the number of seeds that germinated after 1, 2, 3, …, N days from the beginning of inoculation. We only considered AGT and GR as markers of phytotoxicity because these are simple parameters of easy observation. The results were validated through an Analysis of Variance (ANOVA).

### Photosynthetic activity monitoring

**Fig 2:**
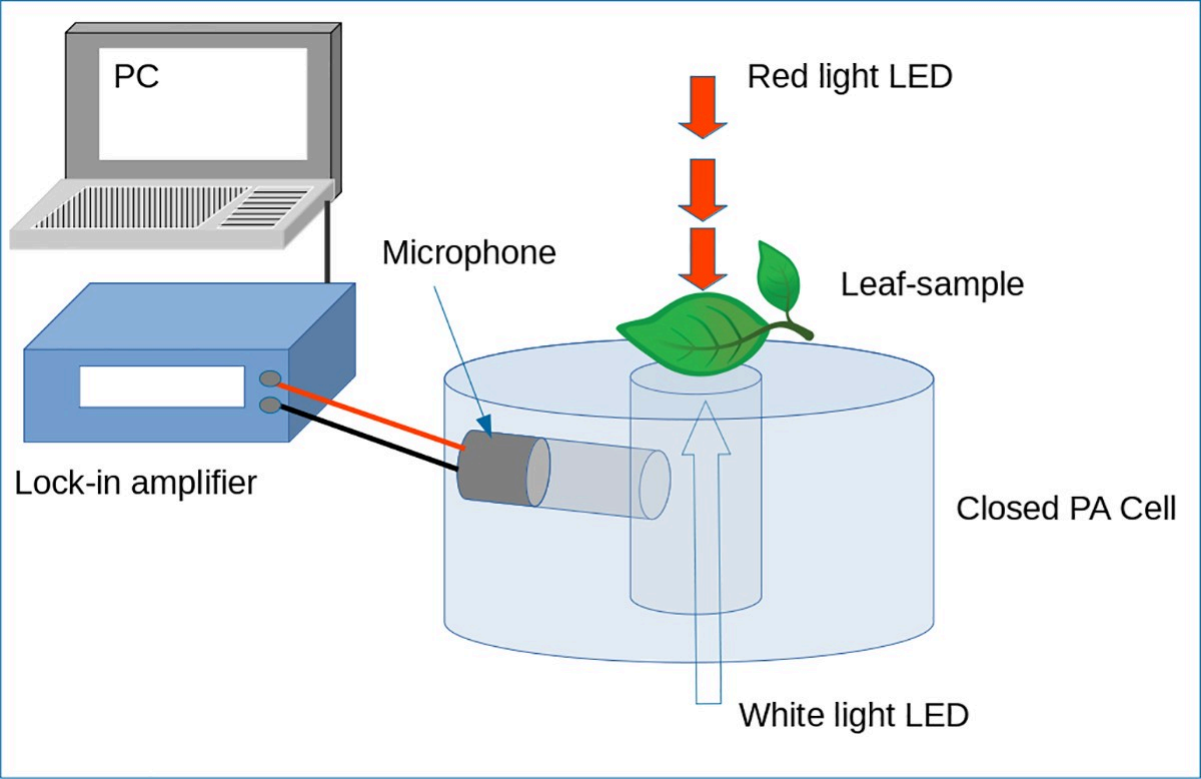
The PA system.

From the group that germinated from the seeds previously treated with Ag-TiO_2_, one plant of each treatment was randomly selected and moved from the greenhouse to the laboratory; there, they were kept in darkness for 30 minutes before taking photosynthetic activity measurements. This procedure was repeated for 20 days. The ratio of oxygen evolution (ROE) was measured using the photoacoustic (PA) technique, a photothermal method in which the sample is periodically impinged with light pulses, whose energy is partially absorbed and transformed into heat. The absorption of this modulated frequency light produces heat and, consequently, periodical changes of pressure in the adjacent air to the sample surface; these variations are detected with a microphone and the analysis of the corresponding signal allows to track the oxygen evolution, generated during the photosynthetic activity, considering the saturation of the photobaric process caused by incidence of continuous light over the leaf [25]. In Fig 2 the PA system is shown, this is composed by two LED, one red and the other white, as sources of modulated and continuous light, respectively. The leaf is placed as the topper of a cavity of a homemade closed PA cell, in which an omni-directional electrect microphone (Radio Shack) is disposed. The amplification of the signal was made through a Lock-in amplifier SR830 (Stanford Research).

## Results and discussion

### Characterization of NPs using X-ray diffraction

**Fig 3:**
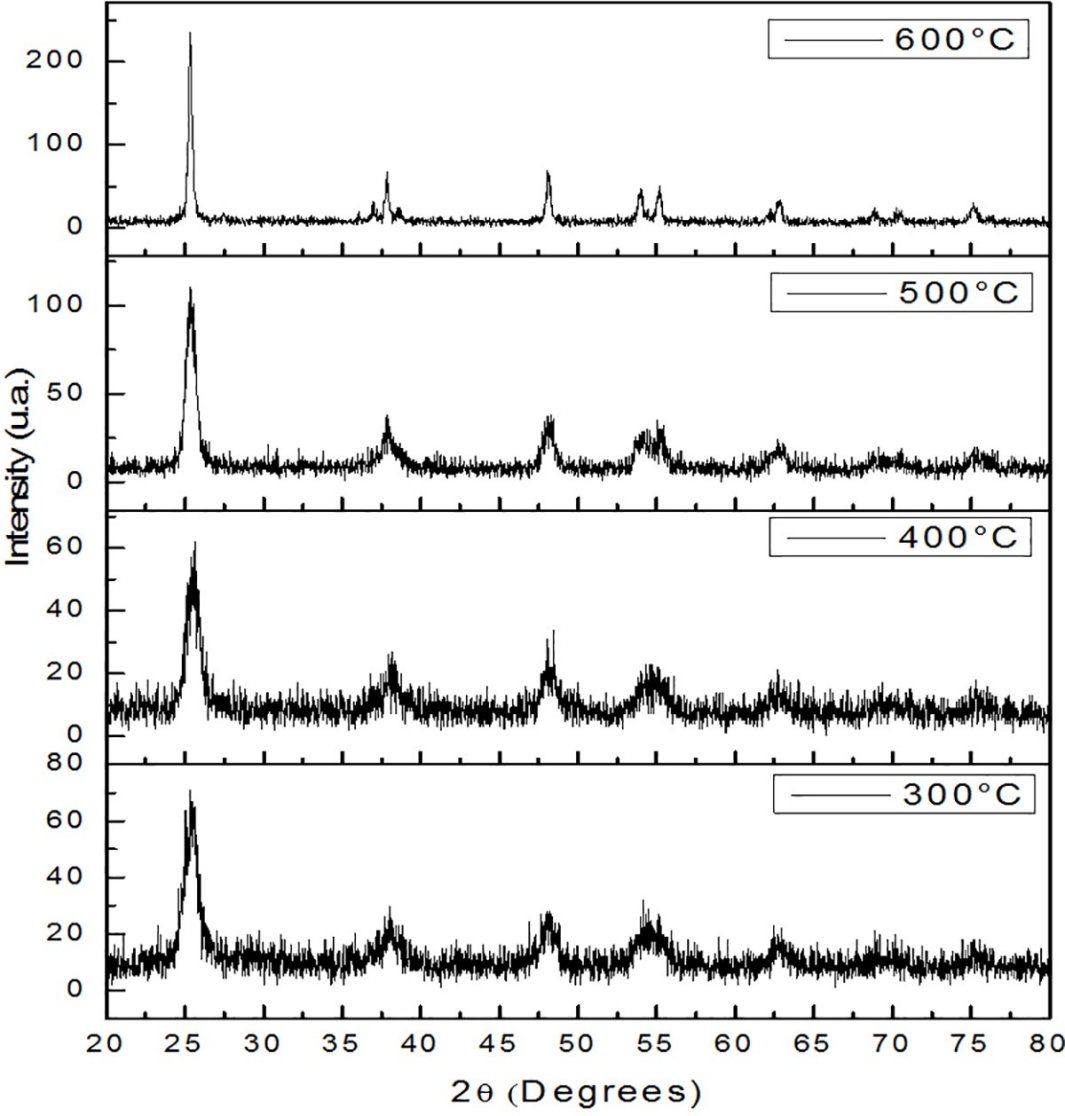
Diffractograms from samples of Ag-TiO_2_. That samples were sintered at 300, 400, 500 and 600°C.

The diffractograms of Ag-TiO_2_ samples are shown in Fig 3; the diffraction peaks centered at 25.4, 37.8, 48.1, 54.6 and 62.8° indicate the presence of anatase phase TiO_2_, corresponding to planes (101), (103), (200), (105) and (204), respectively. With the rise in temperature, the intensity of the peaks increased and their width decreased; this indicates greater crystallinity of the material [26]. The average particle size was estimated from these diffractograms and listed in Table 1.

**Table 1:** Particle sizes of Ag-TiO_2_ powder samples.

Treatment temperature (°C)	Estimated particle size (nm)
300	7.3 ± 0.1
400	8.3 ± 0.4
500	10.9 ± 0.4
600	26.0 ± 0.6

**Fig 4:**
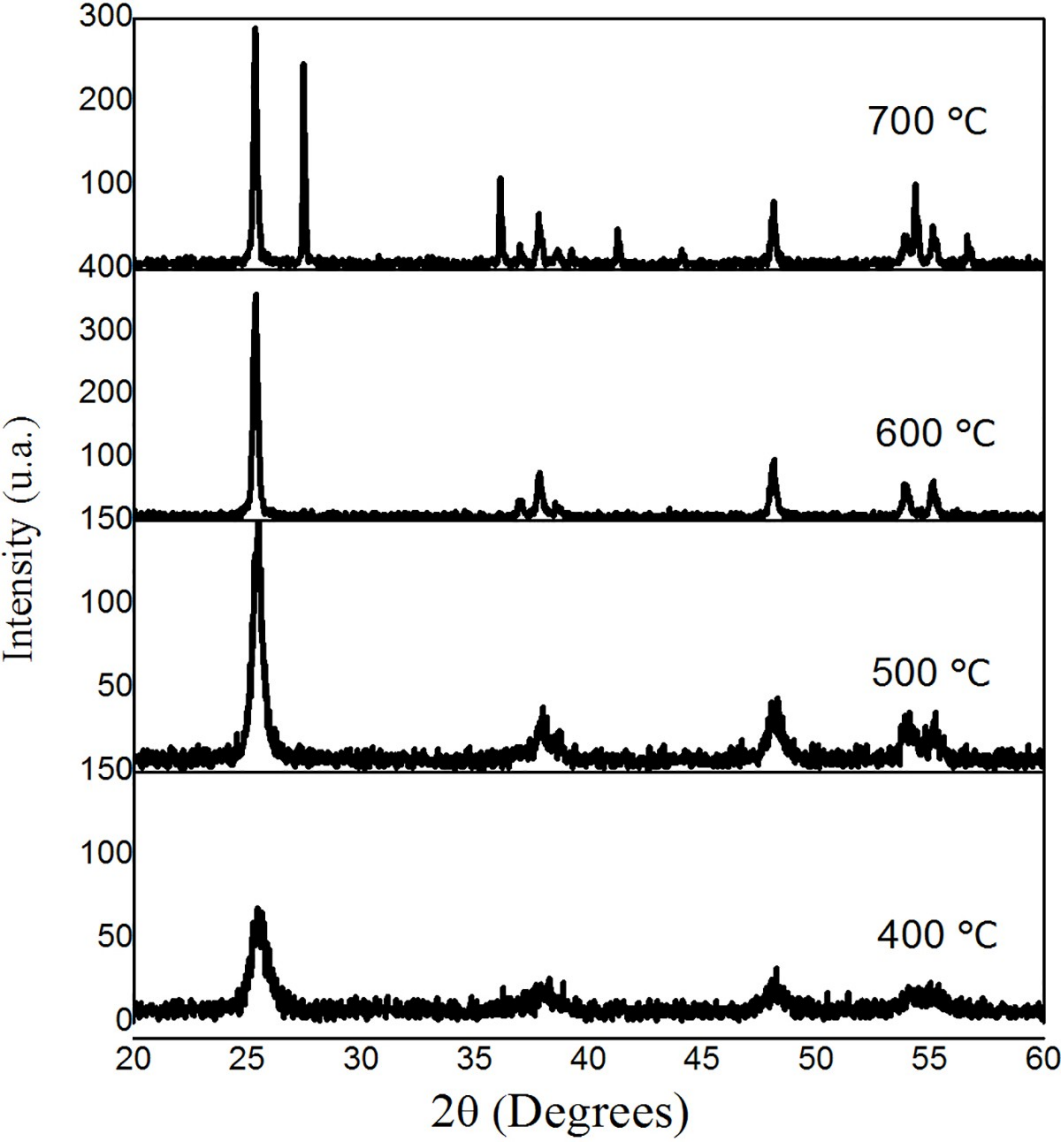
X-ray diffractograms of TiO_2_ powder samples.

In diffractograms from TiO_2_ powder samples, the characteristic peaks of the anatase and rutile phases were observed, as shown in Fig 4. When the temperature of the thermal treatment increased, the crystallinity of the anatase was enhanced, as well as the size of the NPs [27]. Although at 700°C the appearance of a peak in 27.5° from the rutile phase was observed, its weight percentage is negligible in comparison to anatase. Table 2 contains the estimated particle average sizes using Scherrer´s equation [23]:

$$\tau =\frac{K\lambda }{\beta cos(\theta )}$$ (1)

Where *τ* is the particle size in nm; *K =* 0.9 is a shape factor; *λ* = 0.154 nm is the X-ray wavelength; *β* is the line broadening at half of the maximum intensity in radians and *θ* is the Bragg angle.

**Table 2:** Estimated particle sizes of TiO_2_ samples after thermal treatment.

Temperature of thermal treatment (°C)	Estimated particle size (nm)
400	8.8 ± 0.2
500	15.0 ± 0.2
600	32.5 ± 0.6
700	43.1 ± 0.1

Considering that the effect that multiple scattering has on the dynamic light scattering (DLS) technique is enhanced by the increase in concentration, the measurements of hydrodynamic diameter might not be accurate for values higher than 500 mg/L. In this work, this method was not used because the concentrations were higher than this technical limit. Furthermore, it has been reported that for a concentration of 500 mg/L the particle size is not strongly affected by the suspension situation, after sonication [24].

Care was taken to ensure that the after-sonication suspensions were used immediately in the inoculation process and the analysis was done supposing that the sizes in suspension at each concentration were similar to those obtained through this primary characterization of TiO_2_ and Ag-TiO_2_ NPs powder samples.

### Composition of Ag-TiO_2_ powder samples

**Fig 5:**
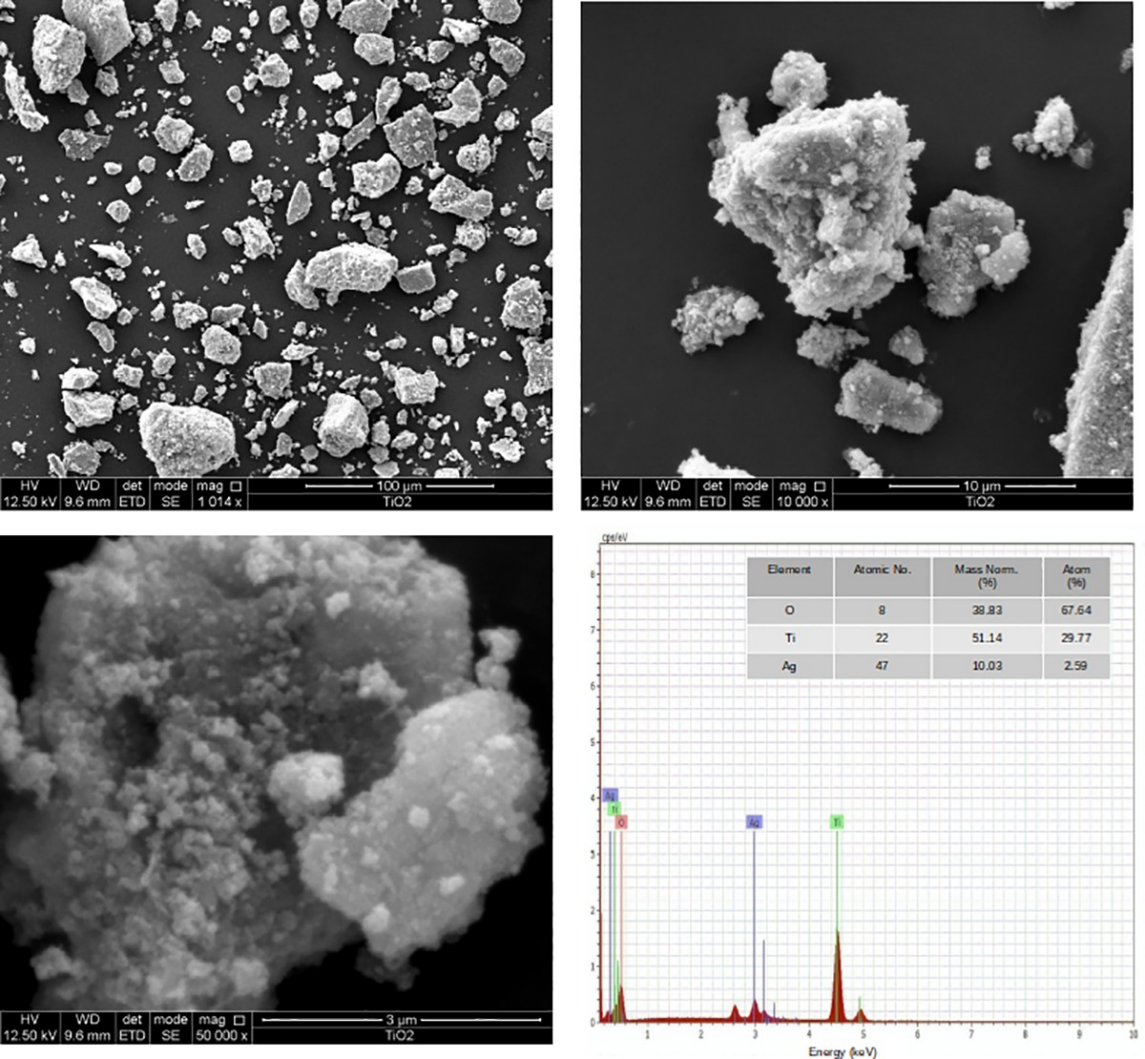
SEM images of the Ag-TiO_2_ sample sintered at 300°C.

The concentration of the elements contained in the nanoparticle material is shown in Table 3; the presence of silver and the main constituents, titanium and oxygen, was confirmed. The signals from these elements indicate that Ag is incorporated into the structure of TiO_2_ [28]. In Fig 5, SEM micrographs of an Ag-TiO_2_ sample (300°C, 7.3 nm) show the common behavior of all samples, highly agglomerated particles with rocklike structures conformed by spherically shaped nanostructures. The EDX graph corresponds to this sample and it confirmed the presence of Ti, Ag and O components.

**Table 3:** Percentage of oxygen, silver and titanium in Ag-TiO_2_ samples, obtained by SEM-EDS.

Element	% Mass	% Atom
Oxygen	36.59	67.64
Titanium	48.19	29.77
Silver	9.45	2.59
Total	94.24	100.00

### Phytotoxicity assessment

Spinach seeds were exposed to suspensions of nanometric particle size TiO_2_ and measurements of germination were examined using multifactorial ANOVA. The P-values proved the statistical significance of each of the factors (particle size and concentration) with a 95% confidence level, which indicates that each of them has a direct effect on the germination of the seeds.

The AGT and GR of the spinach seeds were calculated from the collected data and the resultant values were noted in Table 4. The results of the control group were registered as corresponding to zero particle size and zero concentration, since only distilled water was applied to these plants. At the lowest concentration, of 0.25%, and with the inoculation process of the NPs between 9 and 33 nm, the AGT values were lower than those of the control group and the GR data were higher than those of the control group; while with all used concentrations, with a size of 43 nm, the time to germination lengthened. With 9 nm NPs the lowest AGT of 6.36 days was obtained and it was lower than that corresponding to the control group of 7.14 days, whereas with the NPs smaller than 43 nm, the GR was between 221 and 256% higher than that of the control group.

**Table 4:** Average Germination Time (AGT) and Germination Rate (GR) of spinach seeds, inoculated with the suspension of TiO_2_ powder.

PARTICLE SIZE (nm)	CONCENTRATION (%)	AGT (days)	GR (seeds/day)
0	0	7.14	1.075
9	0,25	6.36	3.46
	2	9.27	1.53
	4	11.12	1.08
	6	11.77	0.78
15	0,25	7	3.50
	2	8.33	2.21
	4	8.87	1.26
	6	8.8	0.93
33	0,25	6.43	3.83
	2	8.6	1.48
	4	11.6	0.45
	6	9	1.27
43	0,25	10	1.32
	2	10.75	1.18
	4	9.81	1.25
	6	9.08	1.50

Feizi *et al*. found similar results [25], they reported a decrease in the AGT of fennel seeds with the exposure to 40 ppm nanometric TiO_2_; the time was 3.99 days, while 4.05 days were registered for the control. Other authors also indicated that the treatment with nanometric size TiO_2_ favored the generation of active oxygen, superoxide anions and hydroxide; this management improved the resistance to stress of the seeds and promoted the absorption of water and oxygen, which accelerates the germination [29].

In the same experiment in which TiO_2_ was applied to fennel seeds [30], the authors reported a 39.5% increase in the GR parameter compared to the control. A similar response was described by other researchers [31], suggesting that the key reason for this increase may be photo-sterilization and oxygen photogeneration due to the photocatalytic action of nanometric TiO_2_. This would improve the resistance to stress of the seeds and the absorption of water and oxygen in germination.

The negative effects of this kind of nanomaterial on plants, associated with high concentration and larger particle size, may be related to the aggregation of clusters that clog the root pores. This interrupts water absorption and leads to an increase of AGT and a decrease of GR [30].

### Morphological analysis of spinach plants

**Fig 6:**
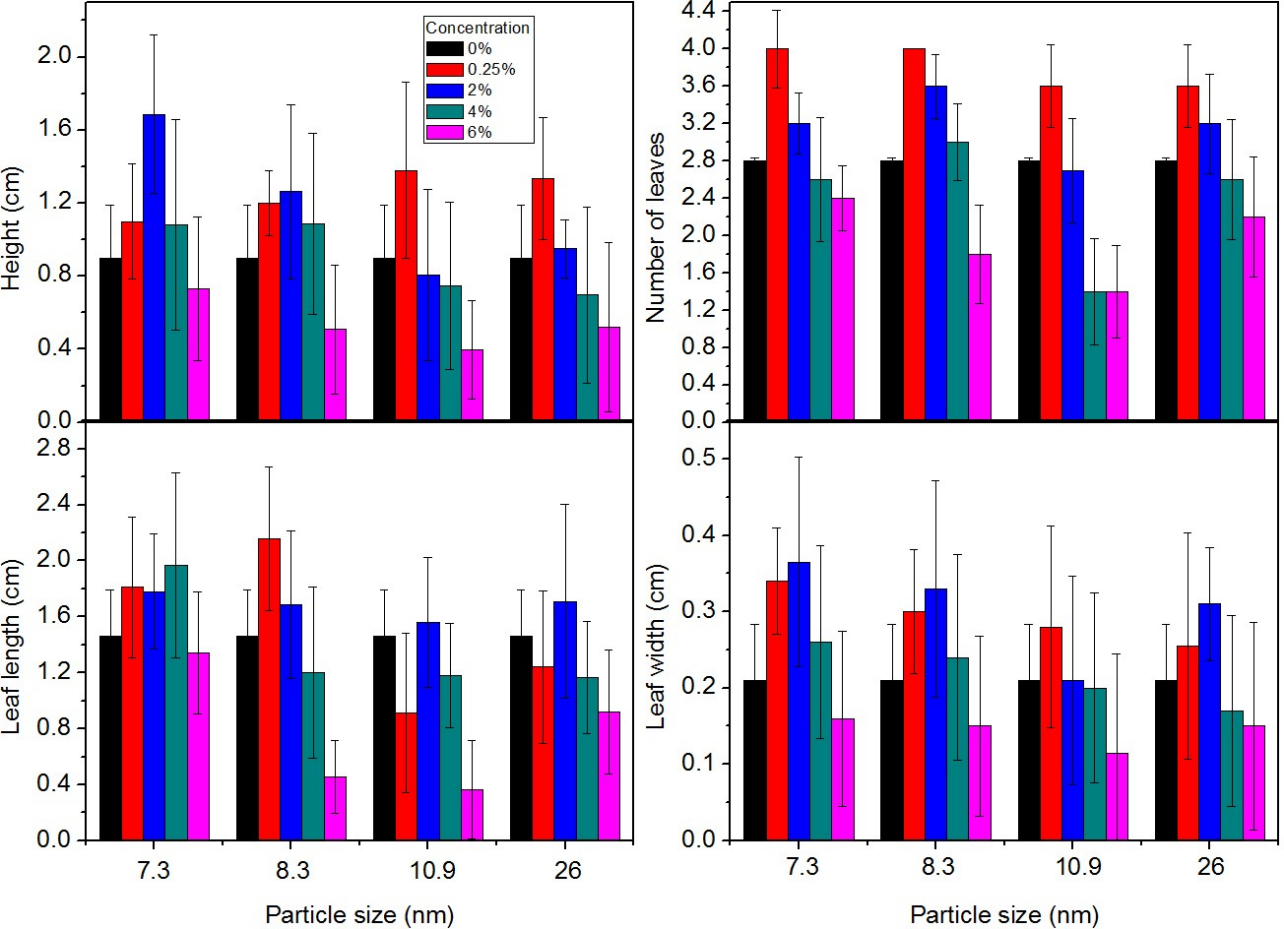
Morphological analysis of spinach plants. Morphological measures of spinach plants 16 days after the Ag-TiO_2_ treatment.

The graphics of multifactorial ANOVA interaction are shown in Fig 6; the particle size and concentration of the Ag-TiO_2_ NPs suspensions were correlated to the morphological parameters of the treated plants as a function of time. From this analysis described in Table 1, the p-values are less than 0.05 for applications of 0.25, 2, 4 and 6% concentrations; this result indicates that the evaluated factors are statistically significant with a confidence level of 95%, and that the development of seedlings is directly affected by the inoculation of the nanomaterial into the seeds.

According to the graphs in Fig 6, the growth parameters, seedling height, leaf dimensions and amount of leaves increased between 30 and 49% for treatments with the suspension of smaller NPs, 7.3 and 8.3 nm, and lower concentrations, 0.25 and 2%, in relation to the average values corresponding to control plants. This might be associated to the regulatory function of TiO_2_ on the activity of enzymes involved in nitrogen metabolism, such as nitrate reductase, glutamate dehydrogenase and glutamine synthase or glutamic-pyruvic transaminase. These enzymes help the plants to absorb nitrates and stimulate the conversion of inorganic nitrogen to organic nitrogen, in the form of proteins and chlorophyll, which increase the weight of the plant and promote its growth and development [32, 33]. Other authors have found that silver NPs have antibacterial and antifungal properties, with effects on germination, root-bud ratio, growth, root lengthening and senescence inhibition [34]. This has been connected with the synthesis of proteins and carbohydrates, the increase of biochemical attributes such as chlorophyll and antioxidant enzymes, the decrease of total phenol contents and with the catalase and peroxidase activities [32].

The behavior of the growth parameters as a function of the concentration and particle size is compared to that of the control group in Fig 6 and the results are consistent with those reported by Lyu *et al*. [32], who argued that the effects of the application of NPs on plants can be positive or negative, depending on their concentration, size and physicochemical properties. This may be related to the increase in reactive oxygen species, which are a hallmark of phytotoxicity [30].

### Photosynthesis monitoring

**Fig 7:**
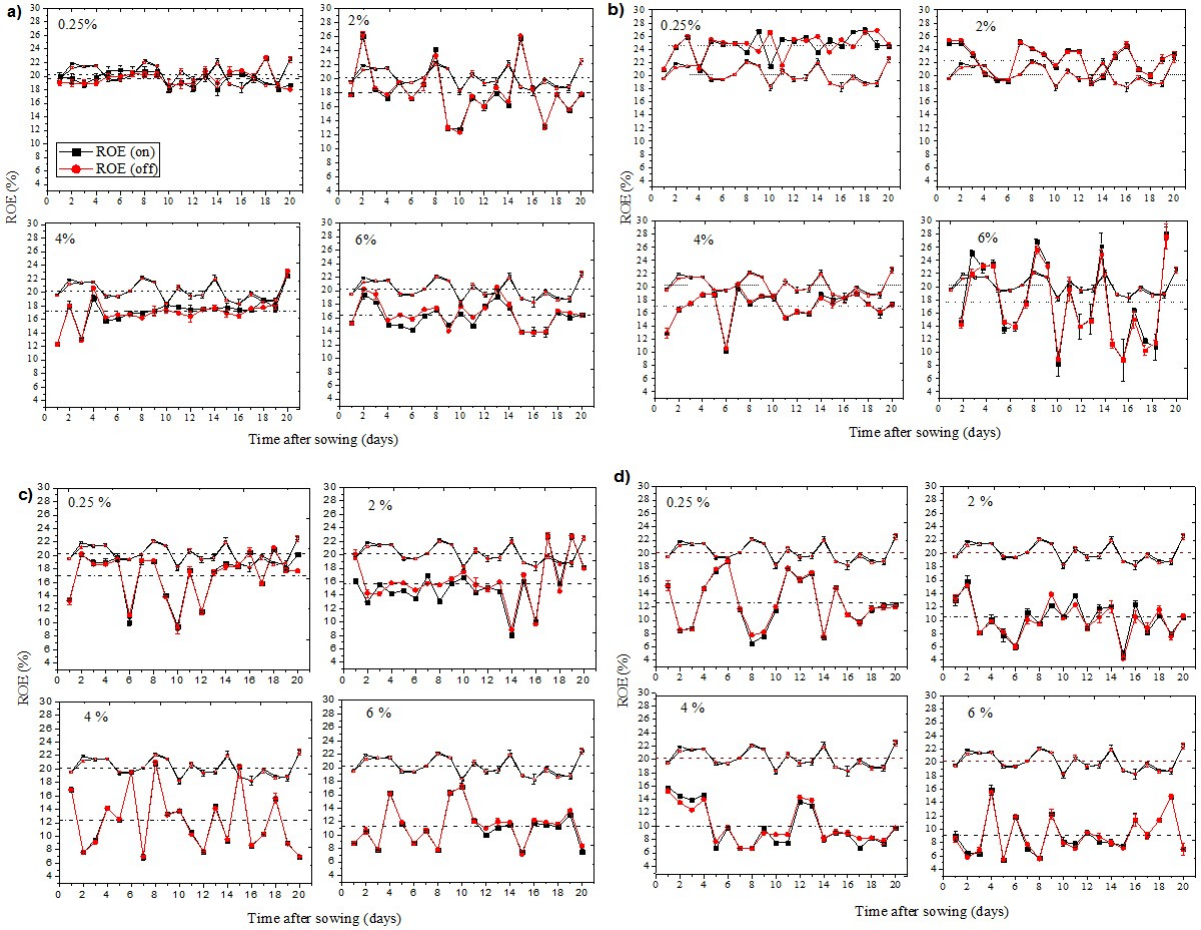
ROE of plants treated with suspensions of TiO_2_-Ag NPs. NPs sizes of a) 7.3 nm, b) 8.3 nm, c) 10.9 nm and d) 26 nm, with 0.25, 2, 4 and 6% concentration.

In Fig 7 the ROE was plotted as a function of time, measured since the sowing of the seedlings that were treated during the germination process with the Ag-TiO_2_ NPs suspension, described in Table 1. In this Figure, the black squares and the red circles correspond to experimental points of ROE measured when the photosynthesis of the plant was saturated (continuous white light on) and after it was restored (continuous white light off), respectively; the empty dots of these same shapes and colors correspond to experimental points from measurements of the control group plants.

In Fig 7, the shape of the curves of ROE as a function of time after sowing indicates a dependence of ROE with the concentration and particle size; it can be seen that only with 8.3 nm size, at 0.25 and 2% concentrations, the corresponding ROE points range around an average value (represented as dotted lines) higher than that of the control group; although this difference was the smallest using a 7.3 nm size at 0.25% concentration. This result could be due to an increase in chlorophyll content caused by the treatment with anatase TiO_2_ NPs, because this nanomaterial could improve the absorption of light and therefore the efficiency of its transformation into electrical and chemical energy during photosynthesis [35]. On the other hand, this argument is also consistent with the possibility that the TiO_2_ NPs could be translocated to vegetative organs such as leaves or stem during the plant's growth [36]. Thus, these NPs could act as a protective filter of the chloroplasts against aging and enhance the photosynthetic assimilation of carbon through the activation of Rubisco (Rubisco and Rubisco activase complex), which promotes carboxylation and favors the growth of plants [37].

Principal components analysis (PCA) is a statistical procedure of variable reduction that creates few components that represent most of the variance in a set of observed variables. It is an unsupervised pattern recognition method for exploratory analysis of data to express them in such a way as to underline their similarities and differences, as described in detail elsewhere [38]. In this work, PCA was used for comparing the ROE values of treated spinach plants.

**Fig 8:**
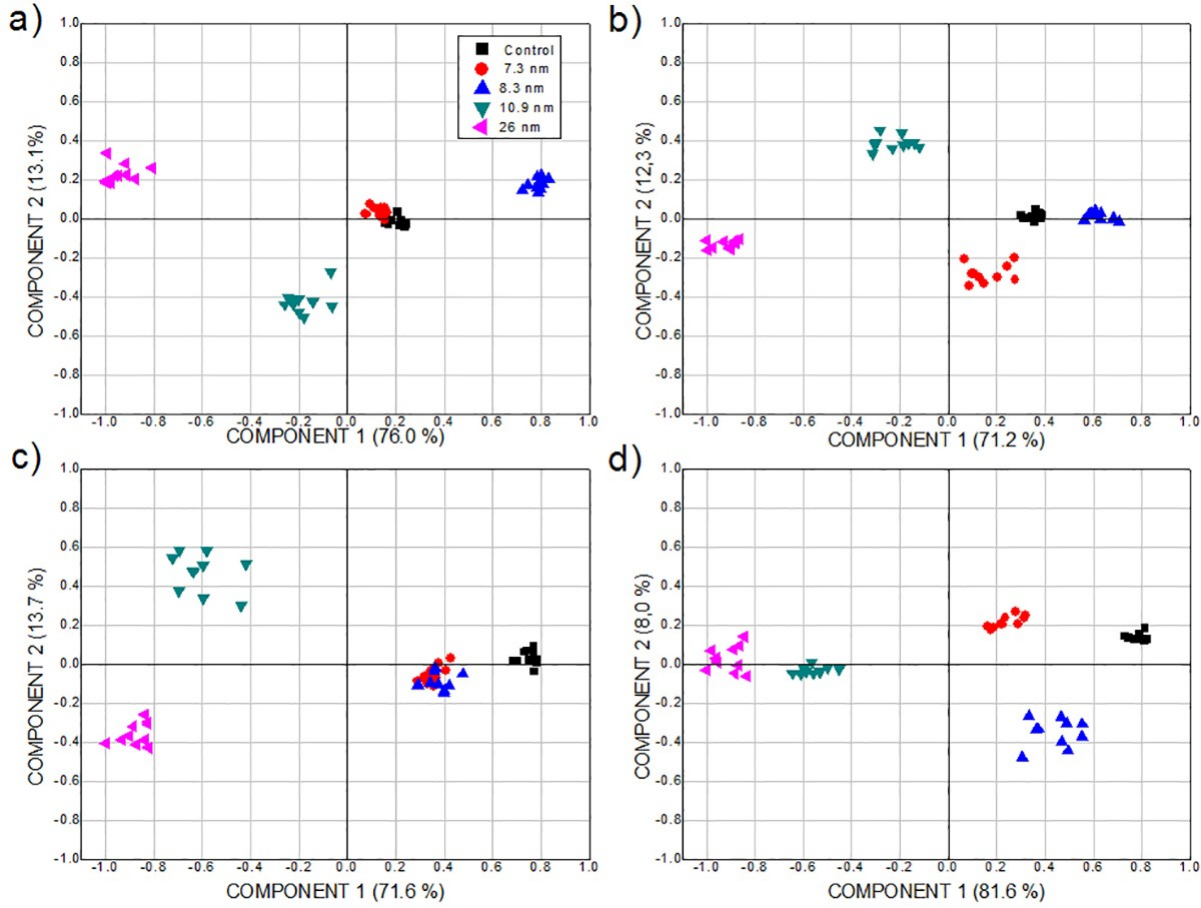
Plots of PCA scores considering the ROE of plants treated with Ag-TiO_2_ NPs. NPs of size 0 (control group), 7.3, 8.3, 10.9 and 26 nm, applying a) 0.25, b) 2, c) 4 and d) 6% concentration.

From the plots of PCA scores, in Fig 8, the main components, PC1 and PC2, explain between 83.5 and 89.6% of the variance. The main component PC1 is related to the concentration of the NPs suspension, while PC2 is associated with the shape of the oscillations of ROE values. PC1 justifies between 71.2 and 81.6% of the variance and PC2 between 8 and 13.7%. At the highest concentration, the variation of the particle size in the suspension has a greater impact on the modification of the ROE behavior of the plants with respect to those of the control group; this is inferred from the proximity of the distribution of points to the PC2 axis. The points furthest from the axis of this component are related to the abrupt variation of the ROE, which corresponds to an oscillation of different shape and greater amplitude than that of the control group. In particular, according to the behavior shown in Fig 8, this stands out for 7.3 and 10.9 nm NPs at 2% concentration and for those of 8.3 nm at 6%.

**Fig 9:**
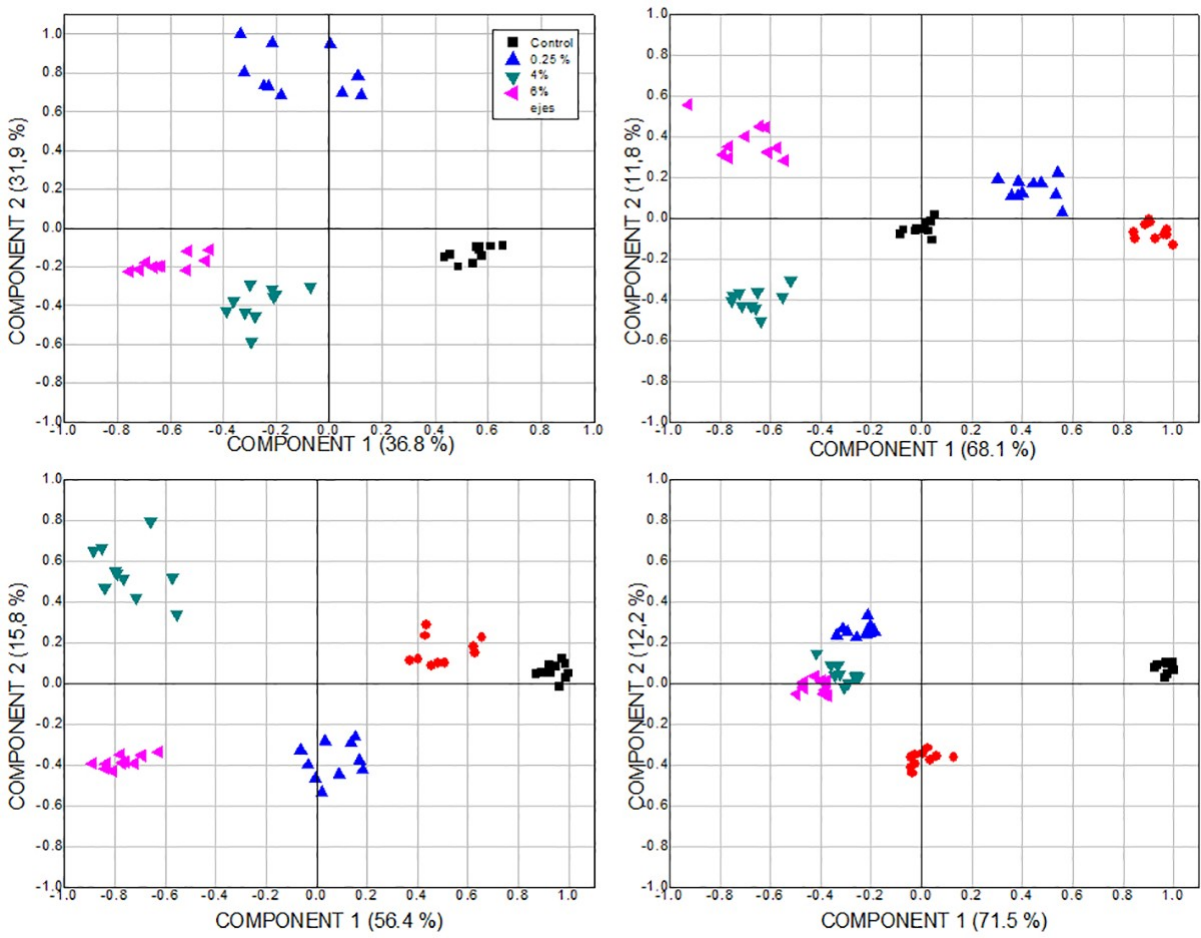
Plots of PCA scores considering the ROE of plants treated with Ag-TiO_2_ NPs. NPs suspension of 0.25, 2, 4 and 6% concentration, of size a) 7.3 nm, b) 8.3 nm, c) 10.9 nm and d) 26 nm.

According to the score graphs of the PCA in Fig 9, the main components PC1 and PC2 justify between 68.7 and 83.7% of the variance. PC1 is related to particle size and explains between 36.8 and 71.5% of the variance; while PC2, which is associated with the shape of the ROE oscillations, between 11.8 and 31.9%. It can be observed that in all cases there is an agglomeration of the corresponding points, which suggests repeatability of ROE behavior depending on the time after the sowing of the plants. For the largest particle size, the change in suspension concentration has a similar effect on the ROE behavior modification regarding the control group. This can be concluded from the proximity of the distribution of points with PC1 and PC2 axis.

The effects of the treatment with Ag-TiO_2_ NPs suspensions on the photosynthetic activity of spinach plants could be associated with the generation of reactive oxygen species, oxidative stress, genotoxic effects, generation of inhibitory enzymes and decrease in cell viability. However, it should be borne in mind that smaller NPs have greater penetration capacity and mobility through plant tissues, while larger particles can be trapped in the roots [16]. In this way, the growth and photosynthetic activity could be disrupted from the accumulation of NPs moving through the shoots of the plants. This could be the cause of the negative morphological results that were obtained with the largest size of the NPs and with the highest concentrations of the suspensions.

Taking into account the control group, the results suggest that a better response to the treatments is obtained when the metabolism of the plant is not drastically altered. The TiO_2_ nanopowder improves spinach growth and chlorophyll formation, and it also increases Rubisco activity as well as the photosynthetic rate during the growth stage, but the effect is inversely proportional to particle size and concentration [36], since the reactive oxygen species produced by high concentrations of nano-TiO_2_ damage the lipidic structure of the membranes, which decreases the photosynthetic capacity [34].

Partial Least Squares–Discriminant Analysis (PLS-DA) does not find hyperplanes of maximum variance between the response and independent variables as PCA, but it projects the predicted and observable variables to a new space, in order to find a linear regression model that allows a supervised pattern recognition method, opening the possibility of making future samples predictions on the modeled classes. A threshold between the predicted and observable values is calculated and the data below this parameter are associated to the distancing of the sample from the modeled class. This threshold splits the classes with the lowest probability of false classifications of future predictions and it is estimated from the distribution of the predicted values obtained through the PLS-DA model through calibration samples [39, 40].

**Fig 10:**
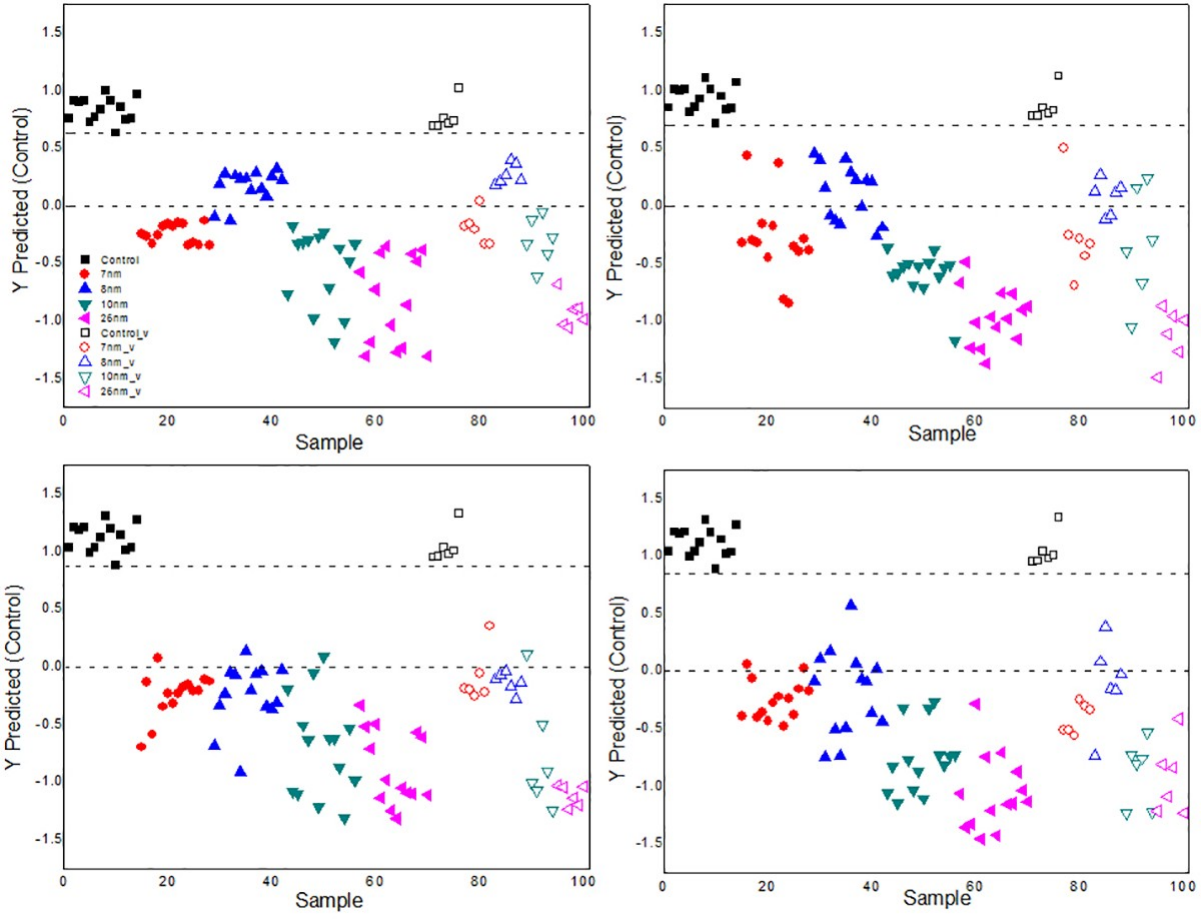
Results of analysis with PLS-DA of the ROE. Behavior of the ROE from plants treated with the NPs and related to the control group. Comparison with class model a) 7 nm, b) 8 nm, c) 10 nm and d) 23 nm. The empty symbols correspond to validation points.

**Fig 11:**
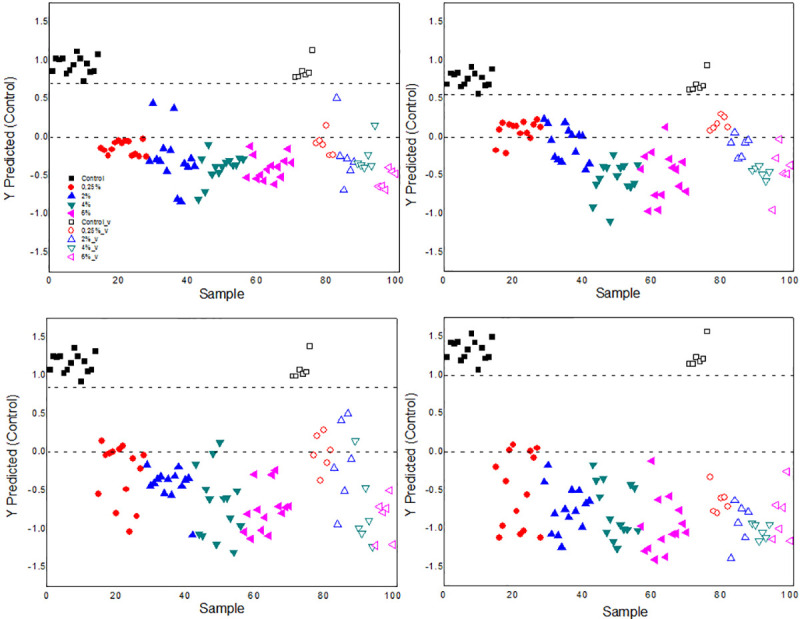
Results of the analysis with PLS-DA of the ROE. Behavior of the ROE from plants treated with the NPs and related to the control group. For the class model of a) 0.25, b) 2, c) 4 and d) 6% concentrations. The empty symbols correspond to the validation points.

The distribution of the class values estimated for the calibration and the validation data sets are shown in Figs 10 and 11 and the separation between them can be observed. The threshold is represented by the upper dotted line. The data corresponds to 40 ROE measurements as a function of time, of 5 plants of each treatment chosen at random with two repetitions for each one, taking into account the calculation made when the light that saturates the photosynthesis process is turned on or off. These curves are selected by a specific algorithm to obtain representative samples for all classes. The control class was modeled versus a large class that contains the data of all samples of the other classes. All control samples were predicted as if they belonged to this same group and none of the samples from the other groups were predicted as control; that is, there were no false positives or false negatives.

For 2% concentration of the suspension, a point corresponding to the size of 7 nm v, predicted as 0 nm appears, and for the 4% point there is a point related to the size of 10 nm v, predicted as 8 nm v. On the other hand, for the suspension with a particle size of 7 nm, a concentration point 2% v was predicted as 0 or 0.25 v; while with 10 nm a point of 4% v was predicted as 2% v.

Both in Figs 10 and 11, it can be seen that, with the model, the prediction closest to the ROE values of the control group corresponds to the application of the NPs suspensions with the smallest particle sizes and lowest concentrations. The threshold increases depending on the rise of the values of these two parameters according to the results obtained by PCA.

## Conclusions

The effect of the inoculation of silver-incorporated titanium dioxide NPs in spinach seeds has a direct dependence on the phytotoxicity, morphology and photosynthetic activity of these plants, depending on the particle size and concentration. In the synthesis of the NPs by sol-gel method, the anatase phase of the titanium dioxide was obtained and the particle size was modified via temperature change in heat treatment. With respect to the control group, the inoculation of the Ag-TiO_2_ NPs of particle sizes between 7.3 and 8.3 nm, at the lowest concentrations of 0.25 and 2%, resulted in the increase of growth parameters, seedling height, leaf dimensions and number of leaves, between 30 and 49%.

According to the estimated ROE, photosynthetic activity increased by 21.78% due to the inoculation of NPs suspensions with 8.3 nm size at 0.25% concentration, taking into account that for the control, the average ROE was between 20.2 and 24.6%. The average germination time was reduced by 10.92% with the treatment of the seeds in the solution of concentration 0.25% and particle size of 9 nm. Although the factors of growth were not improved with the application of the suspension of 33 nm particle size at this same concentration, the germination rate increased at 256.27%.

The treatments that favored the growth of the plants correspond to ROE responses similar to those of the control group. This result was statistically demonstrated through the PCA and the PLS-AD, which explains a high percentage in the variance of the data due to the difference in the characteristics of the applied suspensions and discriminates between the alterations in the photosynthetic activity of the plants.

The best results with Ag-TiO_2_ NPs treatment for spinach plants were obtained with a particle size between 7 and 8 nm at a concentration of 2%. This effect could serve as a starting point to understand the consequences of the inoculation of Ag-TiO_2_ NPs on the physiological and morphological parameters related to plant growth. This information may be useful because these NPs could prevent the exacerbated addition of certain exogenous material to the soil or the environment, which is important for the improvement of crops.

The use of titanium dioxide NPs with silver incorporation could play a role in contemporary agriculture, because the use of these compounds with specific concentrations and particle sizes could be adjusted to the needs of each crop.

## Supporting information

S1 Table Germination monitoring of seeds inoculated with TiO_2_ NPs. (DOCX)

S2 Table Average morphological measurements of spinach plants inoculated with TiO_2_-Ag NPs. (DOCX)

S3 Table ROE data from spinach plants inoculated with 7nm TiO_2_-Ag NPs at different concentrations (S3A 0%, S3B 0.25%, S3C 2%, S3D 4% and S3E 6%). (DOCX)

S4 Table ROE data from spinach plants inoculated with 8nm TiO_2_-Ag NPs at different concentrations (S4A 0%, S4B 0.25%, S4C 2%, S4D 4% and S4E 6%). (DOCX)

S5 Table ROE data from spinach plants inoculated with 10nm TiO_2_-Ag NPs at different concentrations (S5A 0%, S5B 0.25%, S5C 2%, S5D 4% and S5E 6%). (DOCX)

S6 Table ROE data from spinach plants inoculated with 26nm TiO_2_-Ag NPs at different concentrations (S6A 0%, S6B 0.25%, S6C 2%, S6D 4% and S6E 6%). (DOCX)
