# Supplementary material for: Effect of the suspension of Ag-incorporated TiO2 nanoparticles (Ag-TiO2 NPs) on certain growth, physiology and phytotoxicity parameters in spinach seedlings
Source: PLoS One. 2020 Dec 29;15(12):e0244511. doi: 10.1371/journal.pone.0244511 (PMC7771677; doi:10.1371/journal.pone.0244511)
Supplement: S1 Table — (DOCX) [file pone.0244511.s001.docx]

**S1 Table. Germination monitoring of seeds inoculated with TiO_2_ NPs**

| Monitoring day | Particle size (nm) | Concentration (%) | Number of germinated seeds |
| --- | --- | --- | --- |
| 1 | 0 | 0 | 0 |
| 2 | 0 | 0 | 0 |
| 3 | 0 | 0 | 0 |
| 4 | 0 | 0 | 1 |
| 5 | 0 | 0 | 2 |
| 6 | 0 | 0 | 3 |
| 7 | 0 | 0 | 3 |
| 8 | 0 | 0 | 4 |
| 9 | 0 | 0 | 7 |
| 10 | 0 | 0 | 7 |
| 11 | 0 | 0 | 7 |
| 12 | 0 | 0 | 7 |
| 13 | 0 | 0 | 7 |
| 14 | 0 | 0 | 7 |
| 1 | 9 | 0.25 | 0 |
| 2 | 9 | 0.25 | 4 |
| 3 | 9 | 0.25 | 5 |
| 4 | 9 | 0.25 | 5 |
| 5 | 9 | 0.25 | 6 |
| 6 | 9 | 0.25 | 7 |
| 7 | 9 | 0.25 | 7 |
| 8 | 9 | 0.25 | 10 |
| 9 | 9 | 0.25 | 12 |
| 10 | 9 | 0.25 | 12 |
| 11 | 9 | 0.25 | 13 |
| 12 | 9 | 0.25 | 13 |
| 13 | 9 | 0.25 | 13 |
| 14 | 9 | 0.25 | 14 |
| 1 | 15 | 0.25 | 0 |
| 2 | 15 | 0.25 | 3 |
| 3 | 15 | 0.25 | 4 |
| 4 | 15 | 0.25 | 5 |
| 5 | 15 | 0.25 | 5 |
| 6 | 15 | 0.25 | 6 |
| 7 | 15 | 0.25 | 6 |
| 8 | 15 | 0.25 | 12 |
| 9 | 15 | 0.25 | 15 |
| 10 | 15 | 0.25 | 15 |
| 11 | 15 | 0.25 | 16 |
| 12 | 15 | 0.25 | 16 |
| 13 | 15 | 0.25 | 16 |
| 14 | 15 | 0.25 | 17 |
| 1 | 33 | 0.25 | 2 |
| 2 | 33 | 0.25 | 2 |
| 3 | 33 | 0.25 | 2 |
| 4 | 33 | 0.25 | 4 |
| 5 | 33 | 0.25 | 5 |
| 6 | 33 | 0.25 | 5 |
| 7 | 33 | 0.25 | 9 |
| 8 | 33 | 0.25 | 12 |
| 9 | 33 | 0.25 | 13 |
| 10 | 33 | 0.25 | 13 |
| 11 | 33 | 0.25 | 13 |
| 12 | 33 | 0.25 | 13 |
| 13 | 33 | 0.25 | 13 |
| 14 | 33 | 0.25 | 14 |
| 1 | 43 | 0.25 | 0 |
| 2 | 43 | 0.25 | 0 |
| 3 | 43 | 0.25 | 0 |
| 4 | 43 | 0.25 | 1 |
| 5 | 43 | 0.25 | 2 |
| 6 | 43 | 0.25 | 3 |
| 7 | 43 | 0.25 | 4 |
| 8 | 43 | 0.25 | 4 |
| 9 | 43 | 0.25 | 4 |
| 10 | 43 | 0.25 | 5 |
| 11 | 43 | 0.25 | 6 |
| 12 | 43 | 0.25 | 7 |
| 13 | 43 | 0.25 | 8 |
| 14 | 43 | 0.25 | 11 |
| 1 | 9 | 2 | 0 |
| 2 | 9 | 2 | 0 |
| 3 | 9 | 2 | 0 |
| 4 | 9 | 2 | 3 |
| 5 | 9 | 2 | 3 |
| 6 | 9 | 2 | 4 |
| 7 | 9 | 2 | 4 |
| 8 | 9 | 2 | 5 |
| 9 | 9 | 2 | 6 |
| 10 | 9 | 2 | 6 |
| 11 | 9 | 2 | 7 |
| 12 | 9 | 2 | 7 |
| 13 | 9 | 2 | 7 |
| 14 | 9 | 2 | 11 |
| 1 | 15 | 2 | 0 |
| 2 | 15 | 2 | 0 |
| 3 | 15 | 2 | 2 |
| 4 | 15 | 2 | 2 |
| 5 | 15 | 2 | 3 |
| 6 | 15 | 2 | 4 |
| 7 | 15 | 2 | 4 |
| 8 | 15 | 2 | 10 |
| 9 | 15 | 2 | 11 |
| 10 | 15 | 2 | 11 |
| 11 | 15 | 2 | 12 |
| 12 | 15 | 2 | 13 |
| 13 | 15 | 2 | 13 |
| 14 | 15 | 2 | 15 |
| 1 | 33 | 2 | 0 |
| 2 | 33 | 2 | 0 |
| 3 | 33 | 2 | 1 |
| 4 | 33 | 2 | 2 |
| 5 | 33 | 2 | 2 |
| 6 | 33 | 2 | 3 |
| 7 | 33 | 2 | 5 |
| 8 | 33 | 2 | 6 |
| 9 | 33 | 2 | 6 |
| 10 | 33 | 2 | 6 |
| 11 | 33 | 2 | 7 |
| 12 | 33 | 2 | 8 |
| 13 | 33 | 2 | 8 |
| 14 | 33 | 2 | 10 |
| 1 | 43 | 2 | 0 |
| 2 | 43 | 2 | 0 |
| 3 | 43 | 2 | 0 |
| 4 | 43 | 2 | 0 |
| 5 | 43 | 2 | 0 |
| 6 | 43 | 2 | 0 |
| 7 | 43 | 2 | 1 |
| 8 | 43 | 2 | 4 |
| 9 | 43 | 2 | 4 |
| 10 | 43 | 2 | 6 |
| 11 | 43 | 2 | 7 |
| 12 | 43 | 2 | 8 |
| 13 | 43 | 2 | 9 |
| 14 | 43 | 2 | 12 |
| 1 | 9 | 4 | 0 |
| 2 | 9 | 4 | 1 |
| 3 | 9 | 4 | 1 |
| 4 | 9 | 4 | 1 |
| 5 | 9 | 4 | 1 |
| 6 | 9 | 4 | 1 |
| 7 | 9 | 4 | 1 |
| 8 | 9 | 4 | 1 |
| 9 | 9 | 4 | 3 |
| 10 | 9 | 4 | 3 |
| 11 | 9 | 4 | 3 |
| 12 | 9 | 4 | 3 |
| 13 | 9 | 4 | 4 |
| 14 | 9 | 4 | 8 |
| 1 | 15 | 4 | 0 |
| 2 | 15 | 4 | 1 |
| 3 | 15 | 4 | 1 |
| 4 | 15 | 4 | 1 |
| 5 | 15 | 4 | 1 |
| 6 | 15 | 4 | 1 |
| 7 | 15 | 4 | 2 |
| 8 | 15 | 4 | 5 |
| 9 | 15 | 4 | 5 |
| 10 | 15 | 4 | 5 |
| 11 | 15 | 4 | 6 |
| 12 | 15 | 4 | 6 |
| 13 | 15 | 4 | 7 |
| 14 | 15 | 4 | 8 |
| 1 | 33 | 4 | 0 |
| 2 | 33 | 4 | 0 |
| 3 | 33 | 4 | 0 |
| 4 | 33 | 4 | 0 |
| 5 | 33 | 4 | 0 |
| 6 | 33 | 4 | 0 |
| 7 | 33 | 4 | 0 |
| 8 | 33 | 4 | 0 |
| 9 | 33 | 4 | 2 |
| 10 | 33 | 4 | 2 |
| 11 | 33 | 4 | 2 |
| 12 | 33 | 4 | 3 |
| 13 | 33 | 4 | 3 |
| 14 | 33 | 4 | 5 |
| 1 | 43 | 4 | 0 |
| 2 | 43 | 4 | 0 |
| 3 | 43 | 4 | 0 |
| 4 | 43 | 4 | 0 |
| 5 | 43 | 4 | 1 |
| 6 | 43 | 4 | 1 |
| 7 | 43 | 4 | 4 |
| 8 | 43 | 4 | 5 |
| 9 | 43 | 4 | 5 |
| 10 | 43 | 4 | 6 |
| 11 | 43 | 4 | 7 |
| 12 | 43 | 4 | 8 |
| 13 | 43 | 4 | 9 |
| 14 | 43 | 4 | 11 |
| 1 | 9 | 6 | 0 |
| 2 | 9 | 6 | 0 |
| 3 | 9 | 6 | 0 |
| 4 | 9 | 6 | 0 |
| 5 | 9 | 6 | 0 |
| 6 | 9 | 6 | 0 |
| 7 | 9 | 6 | 0 |
| 8 | 9 | 6 | 0 |
| 9 | 9 | 6 | 2 |
| 10 | 9 | 6 | 3 |
| 11 | 9 | 6 | 3 |
| 12 | 9 | 6 | 5 |
| 13 | 9 | 6 | 7 |
| 14 | 9 | 6 | 9 |
| 1 | 15 | 6 | 0 |
| 2 | 15 | 6 | 1 |
| 3 | 15 | 6 | 1 |
| 4 | 15 | 6 | 1 |
| 5 | 15 | 6 | 1 |
| 6 | 15 | 6 | 1 |
| 7 | 15 | 6 | 3 |
| 8 | 15 | 6 | 3 |
| 9 | 15 | 6 | 3 |
| 10 | 15 | 6 | 3 |
| 11 | 15 | 6 | 3 |
| 12 | 15 | 6 | 3 |
| 13 | 15 | 6 | 3 |
| 14 | 15 | 6 | 5 |
| 1 | 33 | 6 | 0 |
| 2 | 33 | 6 | 0 |
| 3 | 33 | 6 | 0 |
| 4 | 33 | 6 | 0 |
| 5 | 33 | 6 | 0 |
| 6 | 33 | 6 | 0 |
| 7 | 33 | 6 | 2 |
| 8 | 33 | 6 | 6 |
| 9 | 33 | 6 | 9 |
| 10 | 33 | 6 | 9 |
| 11 | 33 | 6 | 9 |
| 12 | 33 | 6 | 10 |
| 13 | 33 | 6 | 10 |
| 14 | 33 | 6 | 11 |
| 1 | 43 | 6 | 0 |
| 2 | 43 | 6 | 0 |
| 3 | 43 | 6 | 0 |
| 4 | 43 | 6 | 0 |
| 5 | 43 | 6 | 0 |
| 6 | 43 | 6 | 0 |
| 7 | 43 | 6 | 2 |
| 8 | 43 | 6 | 6 |
| 9 | 43 | 6 | 11 |
| 10 | 43 | 6 | 11 |
| 11 | 43 | 6 | 11 |
| 12 | 43 | 6 | 11 |
| 13 | 43 | 6 | 12 |
| 14 | 43 | 6 | 13 |
