# Supplementary material for: Effect of the suspension of Ag-incorporated TiO2 nanoparticles (Ag-TiO2 NPs) on certain growth, physiology and phytotoxicity parameters in spinach seedlings
Source: PLoS One. 2020 Dec 29;15(12):e0244511. doi: 10.1371/journal.pone.0244511 (PMC7771677; doi:10.1371/journal.pone.0244511)
Supplement: S2 Table — (DOCX) [file pone.0244511.s002.docx]

| **S2 Table. Average morphological measurements of spinach plants inoculated**  **with TiO_2_-Ag NPs.** | | | | | | | | |
| --- | --- | --- | --- | --- | --- | --- | --- | --- |
|  |  |  |  |  |  |  |  |  |
|  |  |  |  |  |  |  |  |  |
| \| Height \| \| \| \| \| \| \| \| \| \| --- \| --- \| --- \| --- \| --- \| --- \| --- \| --- \| --- \| \|  \| 7nm \| Error \| 8nm \| Error \| 10nm \| Error \| 26nm \| Error \| \| 0% \| 0.90 \| 0.29 \| 0.90 \| 0.29 \| 0.90 \| 0.29 \| 0.90 \| 0.29 \| \| 0.25% \| 1.10 \| 0.32 \| 1.20 \| 0.18 \| 1.38 \| 0.48 \| 1.34 \| 0.34 \| \| 2% \| 1.69 \| 0.43 \| 1.26 \| 0.48 \| 0.81 \| 0.47 \| 0.95 \| 0.16 \| \| 4% \| 1.08 \| 0.58 \| 1.09 \| 0.50 \| 0.75 \| 0.46 \| 0.70 \| 0.48 \| \| 6% \| 0.73 \| 0.39 \| 0.51 \| 0.35 \| 0.40 \| 0.27 \| 0.52 \| 0.46 \| \|  \|  \|  \|  \|  \|  \|  \|  \|  \| \| Number of leaves \| \| \| \| \| \| \| \| \| \|  \| 7nm \| Error \| 8nm \| Error \| 10nm \| Error \| 26nm \| Error \| \| 0% \| 2.80 \| 0.03 \| 2.80 \| 0.03 \| 2.80 \| 0.03 \| 2.80 \| 0.03 \| \| 0.25% \| 4.00 \| 0.42 \| 4.00 \| 0.00 \| 3.60 \| 0.44 \| 3.60 \| 0.44 \| \| 2% \| 3.20 \| 0.33 \| 3.60 \| 0.34 \| 2.70 \| 0.56 \| 3.20 \| 0.53 \| \| 4% \| 2.60 \| 0.66 \| 3.00 \| 0.41 \| 1.40 \| 0.57 \| 2.60 \| 0.65 \| \| 6% \| 2.40 \| 0.35 \| 1.80 \| 0.53 \| 1.40 \| 0.50 \| 2.20 \| 0.64 \| \|  \|  \|  \|  \|  \|  \|  \|  \|  \| \| Leaf length \| \| \| \| \| \| \| \| \| \|  \| 7nm \| Error \| 8nm \| Error \| 10nm \| Error \| 26nm \| Error \| \| 0% \| 1.46 \| 0.33 \| 1.46 \| 0.33 \| 1.46 \| 0.33 \| 1.46 \| 0.33 \| \| 0.25% \| 1.81 \| 0.50 \| 2.16 \| 0.51 \| 0.92 \| 0.57 \| 1.24 \| 0.54 \| \| 2% \| 1.78 \| 1.11 \| 1.69 \| 0.53 \| 1.56 \| 0.47 \| 1.71 \| 0.69 \| \| 4% \| 1.97 \| 0.96 \| 1.20 \| 0.61 \| 1.18 \| 0.37 \| 1.17 \| 0.40 \| \| 6% \| 1.34 \| 0.83 \| 0.46 \| 0.26 \| 0.37 \| 0.35 \| 0.92 \| 0.45 \| \|  \|  \|  \|  \|  \|  \|  \|  \|  \| \| Leaf width \| \| \| \| \| \| \| \| \| \|  \| 7nm \| Error \| 8nm \| Error \| 10nm \| Error \| 26nm \| Error \| \| 0% \| 0.21 \| 0.07 \| 0.21 \| 0.07 \| 0.21 \| 0.07 \| 0.21 \| 0.07 \| \| 0.25% \| 0.34 \| 0.07 \| 0.30 \| 0.08 \| 0.28 \| 0.13 \| 0.26 \| 0.15 \| \| 2% \| 0.37 \| 0.14 \| 0.33 \| 0.14 \| 0.21 \| 0.14 \| 0.31 \| 0.07 \| \| 4% \| 0.26 \| 0.13 \| 0.24 \| 0.13 \| 0.20 \| 0.12 \| 0.17 \| 0.13 \| \| 6% \| 0.16 \| 0.11 \| 0.15 \| 0.12 \| 0.12 \| 0.13 \| 0.15 \| 0.14 \| |  |  |  |  |  |  |  |  |
|  |  |  |  |  |  |  |  |  |
