# Supplementary material for: Effect of the suspension of Ag-incorporated TiO2 nanoparticles (Ag-TiO2 NPs) on certain growth, physiology and phytotoxicity parameters in spinach seedlings
Source: PLoS One. 2020 Dec 29;15(12):e0244511. doi: 10.1371/journal.pone.0244511 (PMC7771677; doi:10.1371/journal.pone.0244511)
Supplement: S3 Table — (DOCX) [file pone.0244511.s003.docx]

**S3 Table. ROE data from spinach plants inoculated with 7nm TiO_2_-Ag NPs at different concentrations (S3A 0%, S3B 0.25%, S3C 2%, S3D 4% and S3E 6%).**

**S3A Table. ROE data from spinach plants inoculated with 7 nm TiO_2_-Ag NP at a concentration of 0%.**

| Monitoring days | Negative ROE behavior | Error | Positive ROE behavior | Error |
| --- | --- | --- | --- | --- |
| 1 | 19.61274 | 0.10611 | 19.50663 | 0.13147 |
| 2 | 21.92079 | 0.09424 | 21.23011 | 0.13371 |
| 3 | 21.41876 | 0.16609 | 21.49063 | 0 |
| 4 | 21.55031 | 0.12613 | 21.48577 | 0.01163 |
| 5 | 19.35605 | 0.15002 | 19.58909 | 0.03678 |
| 6 | 19.42259 | 0.15444 | 19.43784 | 0.11114 |
| 7 | 20.24185 | 0.05934 | 20.1859 | 0.12401 |
| 8 | 22.2973 | 0.09153 | 22.02478 | 0.14198 |
| 9 | 21.55031 | 0.01911 | 21.48577 | 0.01163 |
| 10 | 18.15837 | 0.41907 | 18.31637 | 0.05624 |
| 11 | 20.79946 | 0.0252 | 20.69199 | 0.40348 |
| 12 | 19.43348 | 0.4287 | 19.43348 | 0.05235 |
| 13 | 19.6752 | 0.49635 | 19.61885 | 0 |
| 14 | 22.1575 | 0.43054 | 21.8845 | 0.14195 |
| 15 | 18.84017 | 0.15615 | 18.84017 | 0.15356 |
| 16 | 18.28627 | 0.70173 | 18.30624 | 0.06738 |
| 17 | 19.97395 | 0.06069 | 19.66894 | 0.29307 |
| 18 | 18.94019 | 0.08278 | 18.63124 | 0.16307 |
| 19 | 18.72106 | 0.43949 | 18.87797 | 0.08998 |
| 20 | 22.58356 | 0.21981 | 22.47852 | 0.43766 |

**S3B Table. ROE data from spinach plants inoculated with 7 nm TiO_2_-Ag NP at a concentration of 0.25%.**

| Monitoring days | Negative ROE behavior | Error | Positive ROE behavior | Error |
| --- | --- | --- | --- | --- |
| 1 | 19.93205 | 0.58278 | 19.06646 | 0.52615 |
| 2 | 19.66568 | 0.75045 | 18.97861 | 0.45379 |
| 3 | 18.79126 | 0.52351 | 18.96826 | 0.4403 |
| 4 | 19.79126 | 0.52351 | 18.96826 | 0.4403 |
| 5 | 20.7003 | 0.65409 | 19.96205 | 0.36796 |
| 6 | 20.97003 | 0.57082 | 19.92738 | 0.36222 |
| 7 | 20.67707 | 0.67144 | 20.34498 | 0.46183 |
| 8 | 20.72893 | 0.74768 | 20.26879 | 0.44443 |
| 9 | 20.73572 | 0.74692 | 20.27563 | 0.43977 |
| 10 | 18.10251 | 0.57183 | 18.66385 | 0.75292 |
| 11 | 19.17324 | 0.43696 | 18.84868 | 0.72111 |
| 12 | 18.17324 | 0.43696 | 18.84868 | 0.72111 |
| 13 | 20.10962 | 0.94637 | 20.6789 | 0.6705 |
| 14 | 18.08906 | 0.99525 | 18.98721 | 0.6705 |
| 15 | 20.22092 | 0.08448 | 20.6789 | 0.6705 |
| 16 | 20.22092 | 0.14258 | 20.8504 | 0.11913 |
| 17 | 19.68247 | 0.66308 | 19.96205 | 0.36796 |
| 18 | 22.71022 | 0.02351 | 22.78673 | 0.0227 |
| 19 | 18.07435 | 0.02444 | 18.39351 | 0.02733 |
| 20 | 18.71513 | 0.21402 | 18.04001 | 0.08507 |

**S3C Table. ROE data from spinach plants inoculated with 7 nm TiO2-Ag NP at a concentration of 2%.**

| Monitoring days | Negative ROE behavior | Error | Positive ROE behavior | Error |
| --- | --- | --- | --- | --- |
| 1 | 17.82383 | 0.10935 | 17.75326 | 0.06077 |
| 2 | 26.07003 | 0.49495 | 26.3204 | 0.44777 |
| 3 | 18.43361 | 0.17095 | 18.61339 | 0.16953 |
| 4 | 17.17783 | 0.03951 | 17.77465 | 0.08719 |
| 5 | 19.44324 | 0.13239 | 19.4939 | 0.10888 |
| 6 | 17.14684 | 0.08669 | 17.20653 | 0.13825 |
| 7 | 19.17497 | 0.32286 | 19.27408 | 0.95819 |
| 8 | 24.15505 | 0.16566 | 23.29085 | 0.13094 |
| 9 | 12.97311 | 0.27344 | 13.07419 | 0.27344 |
| 10 | 12.89184 | 0.09259 | 12.3658 | 0.08702 |
| 11 | 17.34879 | 0.44659 | 17.54863 | 0.23254 |
| 12 | 16.13553 | 0.67195 | 16.00773 | 0.18054 |
| 13 | 17.98722 | 0.15724 | 18.76171 | 0.19182 |
| 14 | 16.28179 | 0.09111 | 16.77133 | 0.2256 |
| 15 | 25.71731 | 0.22038 | 26.09574 | 0.09028 |
| 16 | 18.71476 | 0.27035 | 18.61777 | 0.18259 |
| 17 | 13.22945 | 0.15797 | 13.20265 | 0.46452 |
| 18 | 17.73011 | 0.11916 | 17.81086 | 0.06573 |
| 19 | 15.52324 | 0.20774 | 15.62285 | 0.03467 |
| 20 | 17.74166 | 0.07898 | 17.83241 | 0.10878 |

**S3D Table. ROE data from spinach plants inoculated with 7 nm TiO2-Ag NP at a concentration of 4%.**

| Monitoring days | Negative ROE behavior | Error | Positive ROE behavior | Error |
| --- | --- | --- | --- | --- |
| 1 | 12.35618 | 0.24241 | 12.39309 | 0.30726 |
| 2 | 18.09948 | 0.62538 | 17.98729 | 0.24236 |
| 3 | 13.12758 | 0.14802 | 12.96204 | 0.37505 |
| 4 | 19.19793 | 0.45753 | 20.61843 | 0.2749 |
| 5 | 15.82545 | 0.2571 | 16.2783 | 0.07772 |
| 6 | 16.08569 | 0.12269 | 16.6844 | 0.21936 |
| 7 | 16.87949 | 0.31853 | 16.6844 | 0.25457 |
| 8 | 16.99558 | 0.10856 | 16.2492 | 0.15792 |
| 9 | 17.29321 | 0.65596 | 17.20642 | 0.7047 |
| 10 | 18.13704 | 0.44757 | 17.3585 | 0.46467 |
| 11 | 17.85298 | 0.14303 | 16.91388 | 0.11403 |
| 12 | 17.43822 | 0.61932 | 16.43513 | 0.82134 |
| 13 | 17.53324 | 0.15724 | 17.46119 | 0.19182 |
| 14 | 17.67542 | 0.44254 | 17.67542 | 0.50108 |
| 15 | 17.78803 | 0.66776 | 16.89393 | 0.52007 |
| 16 | 17.49469 | 0.76133 | 16.54531 | 0.31564 |
| 17 | 17.37246 | 0.13685 | 17.63381 | 0.19084 |
| 18 | 18.86471 | 0.11916 | 17.75394 | 0.06573 |
| 19 | 17.71627 | 0.69235 | 18.21042 | 0.62821 |
| 20 | 22.52719 | 0.21458 | 23.16384 | 0.1731 |

**S3E Table. ROE data from spinach plants inoculated with 7 nm TiO_2_-Ag NP at a concentration of 6%.**

| Monitoring days | Negative ROE behavior | Error | Positive ROE behavior | Error |
| --- | --- | --- | --- | --- |
| 1 | 15.3052 | 0.2162 | 15.17354 | 0.15162 |
| 2 | 19.48956 | 0.72322 | 20.20173 | 0.25787 |
| 3 | 18.33213 | 0.20634 | 19.45149 | 0.68963 |
| 4 | 14.92939 | 0.08702 | 15.63878 | 0.08719 |
| 5 | 14.83798 | 0.06218 | 16.43136 | 0.0614 |
| 6 | 14.18992 | 0.08669 | 15.79542 | 0.03951 |
| 7 | 16.2315 | 0.08891 | 17.20723 | 0.13604 |
| 8 | 17.14129 | 0.16566 | 17.47066 | 0.13094 |
| 9 | 14.92939 | 0.0614 | 14.06733 | 0.07087 |
| 10 | 16.65232 | 0.26401 | 17.69821 | 0.3318 |
| 11 | 14.8725 | 0.26401 | 16.05837 | 0.11403 |
| 12 | 17.71666 | 0.11325 | 17.46045 | 0.13037 |
| 13 | 19.14945 | 0.38717 | 20.48549 | 0.39518 |
| 14 | 17.45118 | 0.08515 | 17.9265 | 0.38717 |
| 15 | 13.95769 | 0.22038 | 13.88422 | 0.09028 |
| 16 | 13.94726 | 0.64511 | 13.80304 | 0.08251 |
| 17 | 13.89144 | 0.76133 | 13.95769 | 0.14762 |
| 18 | 16.86166 | 0.2256 | 17.03921 | 0.09867 |
| 19 | 15.99933 | 0.11081 | 16.68538 | 0.0778 |
| 20 | 16.44521 | 0.25182 | 16.33654 | 0.2256 |
