# Supplementary material for: Effect of the suspension of Ag-incorporated TiO2 nanoparticles (Ag-TiO2 NPs) on certain growth, physiology and phytotoxicity parameters in spinach seedlings
Source: PLoS One. 2020 Dec 29;15(12):e0244511. doi: 10.1371/journal.pone.0244511 (PMC7771677; doi:10.1371/journal.pone.0244511)
Supplement: S4 Table — (DOCX) [file pone.0244511.s004.docx]

**S4 Table. ROE data from spinach plants inoculated with 8nm TiO2-Ag NPs at different concentrations (S4A 0%, S4B 0.25%, S4C 2%, S4D 4% and S4E 6%).**

**S4A Table. ROE data from spinach plants inoculated with 8 nm TiO2-Ag NP at a concentration of 0%.**

| Monitoring days | Negative ROE behavior | Error | Positive ROE behavior | Error |
| --- | --- | --- | --- | --- |
| 1 | 19.61274 | 0.10611 | 19.50663 | 0.13147 |
| 2 | 21.92079 | 0.09424 | 21.23011 | 0.13371 |
| 3 | 21.41876 | 0.16609 | 21.49063 | 0 |
| 4 | 21.55031 | 0.12613 | 21.48577 | 0.01163 |
| 5 | 19.35605 | 0.15002 | 19.58909 | 0.03678 |
| 6 | 19.42259 | 0.15444 | 19.43784 | 0.11114 |
| 7 | 20.24185 | 0.05934 | 20.1859 | 0.12401 |
| 8 | 22.2973 | 0.09153 | 22.02478 | 0.14198 |
| 9 | 21.55031 | 0.01911 | 21.48577 | 0.01163 |
| 10 | 18.15837 | 0.41907 | 18.31637 | 0.05624 |
| 11 | 20.79946 | 0.0252 | 20.69199 | 0.40348 |
| 12 | 19.43348 | 0.4287 | 19.43348 | 0.05235 |
| 13 | 19.6752 | 0.49635 | 19.61885 | 0 |
| 14 | 22.1575 | 0.43054 | 21.8845 | 0.14195 |
| 15 | 18.84017 | 0.15615 | 18.84017 | 0.15356 |
| 16 | 18.28627 | 0.70173 | 18.30624 | 0.06738 |
| 17 | 19.97395 | 0.06069 | 19.66894 | 0.29307 |
| 18 | 18.94019 | 0.08278 | 18.63124 | 0.16307 |
| 19 | 18.72106 | 0.43949 | 18.87797 | 0.08998 |
| 20 | 22.58356 | 0.21981 | 22.47852 | 0.43766 |

**S4B Table. ROE data from spinach plants inoculated with 8 nm TiO2-Ag NP at a concentration of 0.25%.**

| Monitoring days | Negative ROE behavior | Error | Positive ROE behavior | Error |
| --- | --- | --- | --- | --- |
| 1 | 20.90799 | 0.04295 | 20.9854 | 0.14978 |
| 2 | 24.29287 | 0.13626 | 24.37019 | 0.08923 |
| 3 | 25.91122 | 0.04861 | 25.96688 | 0.22219 |
| 4 | 20.75104 | 0.07594 | 21.06898 | 0.15374 |
| 5 | 25.28165 | 0.04861 | 25.45815 | 0.22259 |
| 6 | 24.78293 | 0.27159 | 25.00408 | 0.22259 |
| 7 | 24.94026 | 0.27159 | 24.88443 | 0.22259 |
| 8 | 23.57481 | 0.27159 | 24.88443 | 0.07594 |
| 9 | 26.72883 | 0.11229 | 23.67659 | 0.02408 |
| 10 | 21.44026 | 0.2553 | 26.55128 | 0.2553 |
| 11 | 25.56928 | 0.13847 | 21.56842 | 0.14199 |
| 12 | 25.15747 | 0.16869 | 25.53989 | 0.14045 |
| 13 | 25.91122 | 0.09634 | 25.31624 | 0.13783 |
| 14 | 23.54879 | 0.16314 | 25.96688 | 0.16293 |
| 15 | 25.37843 | 0.4658 | 23.55189 | 0.24419 |
| 16 | 24.44855 | 0.24176 | 25.44839 | 0.18165 |
| 17 | 26.68665 | 0.04861 | 24.37064 | 0.22219 |
| 18 | 26.96675 | 0.34991 | 26.59275 | 0.27159 |
| 19 | 24.65656 | 0.64116 | 26.86575 | 0.11229 |
| 20 | 24.53194 | 0.13831 | 24.7422 | 0.2553 |

**S4C Table. ROE data from spinach plants inoculated with 8 nm TiO2-Ag NP at a concentration of 2%.**

| Monitoring days | Negative ROE behavior | Error | Positive ROE behavior | Error |
| --- | --- | --- | --- | --- |
| 1 | 24.94225 | 0.10877 | 25.38033 | 0.0976 |
| 2 | 24.94225 | 0.17095 | 25.38033 | 0.12225 |
| 3 | 23.05895 | 0.17095 | 23.34689 | 0.27158 |
| 4 | 20.23571 | 0.13986 | 20.39303 | 0.19989 |
| 5 | 19.29665 | 0.10877 | 19.37358 | 0.0976 |
| 6 | 19.14679 | 0.17095 | 19.20329 | 0.18969 |
| 7 | 25.09928 | 0.32286 | 25.05278 | 0.08481 |
| 8 | 24.12368 | 0.16566 | 24.18394 | 0.24123 |
| 9 | 23.17556 | 0.20516 | 23.31095 | 0.21212 |
| 10 | 21.1768 | 0.06667 | 21.55129 | 0.17939 |
| 11 | 23.93398 | 0.07855 | 23.62897 | 0.23254 |
| 12 | 23.80009 | 0.05479 | 23.60293 | 0.18054 |
| 13 | 18.74641 | 0.06667 | 18.83705 | 0.19182 |
| 14 | 19.667 | 0.35813 | 20.03839 | 0.30799 |
| 15 | 22.83895 | 0.29668 | 23.17056 | 0.53586 |
| 16 | 24.48842 | 0.18308 | 24.73267 | 0.42193 |
| 17 | 20.94238 | 0.39409 | 20.91326 | 0.08201 |
| 18 | 19.92413 | 0.45621 | 19.96569 | 0.30481 |
| 19 | 22.50913 | 0.20774 | 22.54935 | 0.6829 |
| 20 | 23.45566 | 0.07898 | 23.41398 | 0.29996 |

**S4D Table. ROE data from spinach plants inoculated with 8 nm TiO2-Ag NP at a concentration of 4%.**

| Monitoring days | Negative ROE behavior | Error | Positive ROE behavior | Error |
| --- | --- | --- | --- | --- |
| 1 | 12.87506 | 0.07087 | 12.89738 | 0.72322 |
| 2 | 16.48337 | 0.3318 | 16.51255 | 0.20634 |
| 3 | 17.48454 | 0.11403 | 17.48454 | 0.08702 |
| 4 | 18.80836 | 0.13037 | 18.8272 | 0.06218 |
| 5 | 18.806 | 0.39518 | 18.80034 | 0.08669 |
| 6 | 10.24467 | 0.38717 | 10.64302 | 0.08891 |
| 7 | 19.7399 | 0.09028 | 20.3832 | 0.16566 |
| 8 | 17.31693 | 0.08251 | 17.68389 | 0.0614 |
| 9 | 18.5177 | 0.14762 | 18.60531 | 0.26401 |
| 10 | 18.3813 | 0.09867 | 18.62474 | 0.26401 |
| 11 | 15.19821 | 0.0778 | 15.26728 | 0.11325 |
| 12 | 16.11324 | 0.2256 | 16.20026 | 0.38717 |
| 13 | 15.85605 | 0.15724 | 15.95194 | 0.08515 |
| 14 | 18.7945 | 0.44254 | 18.23199 | 0.22038 |
| 15 | 18.09519 | 0.66776 | 17.37917 | 0.64511 |
| 16 | 18.32038 | 0.76133 | 18.23913 | 0.76133 |
| 17 | 18.90752 | 0.13685 | 18.95544 | 0.2256 |
| 18 | 17.39285 | 0.11916 | 17.27217 | 0.11081 |
| 19 | 15.95793 | 0.69235 | 16.20163 | 0.25182 |
| 20 | 17.41123 | 0.21458 | 17.18641 | 0.1731 |

**S4E Table. ROE data from spinach plants inoculated with 8 nm TiO2-Ag NP at a concentration of 6%.**

| Monitoring days | Negative ROE behavior | Error | Positive ROE behavior | Error |
| --- | --- | --- | --- | --- |
| 1 | 19.36799 | 0.2162 | 17.64163 | 0.15162 |
| 2 | 19.28499 | 0.72322 | 19.36899 | 0.25787 |
| 3 | 11.58676 | 0.20634 | 11.58676 | 0.68963 |
| 4 | 21.37592 | 0.08702 | 21.24247 | 0.08719 |
| 5 | 13.76286 | 0.06218 | 13.78759 | 0.0614 |
| 6 | 12.29151 | 0.08669 | 11.87718 | 0.03951 |
| 7 | 13.52626 | 0.08891 | 13.52626 | 0.13604 |
| 8 | 23.63547 | 0.16566 | 23.44368 | 0.13094 |
| 9 | 19.6807 | 0.0614 | 19.90118 | 0.07087 |
| 10 | 18.94088 | 0.26401 | 18.99216 | 0.3318 |
| 11 | 18.50104 | 0.26401 | 18.52756 | 0.11403 |
| 12 | 16.97149 | 0.11325 | 16.99778 | 0.13037 |
| 13 | 19.69912 | 0.38717 | 19.79669 | 0.39518 |
| 14 | 15.99422 | 0.08515 | 15.91643 | 0.38717 |
| 15 | 12.34513 | 0.22038 | 12.4233 | 0.09028 |
| 16 | 19.40607 | 0.64511 | 19.49108 | 0.08251 |
| 17 | 23.14863 | 0.76133 | 23.38668 | 0.14762 |
| 18 | 17.46032 | 0.2256 | 17.66744 | 0.09867 |
| 19 | 17.17452 | 0.11081 | 16.85318 | 0.0778 |
| 20 | 18.76304 | 0.25182 | 18.95839 | 0.2256 |
