# Supplementary material for: Effect of the suspension of Ag-incorporated TiO2 nanoparticles (Ag-TiO2 NPs) on certain growth, physiology and phytotoxicity parameters in spinach seedlings
Source: PLoS One. 2020 Dec 29;15(12):e0244511. doi: 10.1371/journal.pone.0244511 (PMC7771677; doi:10.1371/journal.pone.0244511)
Supplement: S5 Table — (DOCX) [file pone.0244511.s005.docx]

**S5 Table. ROE data from spinach plants inoculated with 10nm TiO2-Ag NPs at different concentrations (S5A 0%, S5B 0.25%, S5C 2%, S5D 4% and S5E 6%).**

**S5A Table. ROE data from spinach plants inoculated with 10 nm TiO2-Ag NP at a concentration of 0%.**

| Monitoring days | Negative ROE behavior | Error | Positive ROE behavior | Error |
| --- | --- | --- | --- | --- |
| 1 | 19.61274 | 0.10611 | 19.50663 | 0.13147 |
| 2 | 21.92079 | 0.09424 | 21.23011 | 0.13371 |
| 3 | 21.41876 | 0.16609 | 21.49063 | 0 |
| 4 | 21.55031 | 0.12613 | 21.48577 | 0.01163 |
| 5 | 19.35605 | 0.15002 | 19.58909 | 0.03678 |
| 6 | 19.42259 | 0.15444 | 19.43784 | 0.11114 |
| 7 | 20.24185 | 0.05934 | 20.1859 | 0.12401 |
| 8 | 22.2973 | 0.09153 | 22.02478 | 0.14198 |
| 9 | 21.55031 | 0.01911 | 21.48577 | 0.01163 |
| 10 | 18.15837 | 0.41907 | 18.31637 | 0.05624 |
| 11 | 20.79946 | 0.0252 | 20.69199 | 0.40348 |
| 12 | 19.43348 | 0.4287 | 19.43348 | 0.05235 |
| 13 | 19.6752 | 0.49635 | 19.61885 | 0 |
| 14 | 22.1575 | 0.43054 | 21.8845 | 0.14195 |
| 15 | 18.84017 | 0.15615 | 18.84017 | 0.15356 |
| 16 | 18.28627 | 0.70173 | 18.30624 | 0.06738 |
| 17 | 19.97395 | 0.06069 | 19.66894 | 0.29307 |
| 18 | 18.94019 | 0.08278 | 18.63124 | 0.16307 |
| 19 | 18.72106 | 0.43949 | 18.87797 | 0.08998 |
| 20 | 22.58356 | 0.21981 | 22.47852 | 0.43766 |

**S5B Table. ROE data from spinach plants inoculated with 10 nm TiO2-Ag NP at a concentration of 0.25%.**

| Monitoring days | Negative ROE behavior | Error | Positive ROE behavior | Error |
| --- | --- | --- | --- | --- |
| 1 | 13.40152 | 0.13773 | 13.25852 | 0.52615 |
| 2 | 20.10602 | 0.21407 | 20.22684 | 0.10611 |
| 3 | 18.94112 | 0.42727 | 18.68132 | 0.09424 |
| 4 | 18.94112 | 0.52351 | 18.68132 | 0.16609 |
| 5 | 19.6803 | 0.65409 | 19.31975 | 0.12613 |
| 6 | 10.12033 | 0.57082 | 11.09911 | 0.15002 |
| 7 | 19.26901 | 0.67144 | 19.26901 | 0.15444 |
| 8 | 19.11479 | 0.16609 | 19.21411 | 0.05934 |
| 9 | 14.02506 | 0.12613 | 13.83688 | 0.09153 |
| 10 | 9.5071 | 0.49635 | 9.06047 | 0.75292 |
| 11 | 17.71776 | 0 | 17.58213 | 0.72111 |
| 12 | 11.62071 | 0.14195 | 11.6557 | 0.4287 |
| 13 | 17.71175 | 0.15356 | 17.44313 | 0.49635 |
| 14 | 18.82546 | 0.06738 | 18.31093 | 0.43054 |
| 15 | 18.36258 | 0.29307 | 18.3738 | 0.15615 |
| 16 | 20.46058 | 0.16307 | 20.51539 | 0.70173 |
| 17 | 15.90481 | 0.08998 | 15.83495 | 0.06069 |
| 18 | 20.93969 | 0.02351 | 21.16575 | 0.08278 |
| 19 | 17.91746 | 0.02444 | 17.8591 | 0.43949 |
| 20 | 20.12116 | 0.21402 | 17.69852 | 0.21981 |

**S5C Table. ROE data from spinach plants inoculated with 10 nm TiO2-Ag NP at a concentration of 2%.**

| Monitoring days | Negative ROE behavior | Error | Positive ROE behavior | Error |
| --- | --- | --- | --- | --- |
| 1 | 16.20613 | 0.01163 | 20.03547 | 0.70415 |
| 2 | 12.98726 | 0.03678 | 14.27632 | 0.59375 |
| 3 | 15.54544 | 0.11114 | 14.22599 | 0.29098 |
| 4 | 14.26583 | 0.12401 | 15.78967 | 0.12401 |
| 5 | 14.7025 | 0.14198 | 15.80786 | 0.19167 |
| 6 | 13.51381 | 0.01163 | 14.74196 | 0.08003 |
| 7 | 16.91802 | 0.05235 | 15.75346 | 0.05297 |
| 8 | 13.11559 | 0.40348 | 15.49673 | 0.05235 |
| 9 | 15.76556 | 0.05235 | 16.44961 | 0.1589 |
| 10 | 16.63282 | 0 | 17.45629 | 0.17818 |
| 11 | 14.46448 | 0.14195 | 15.50909 | 1.01188 |
| 12 | 15.22736 | 0.20385 | 14.87355 | 0.43766 |
| 13 | 14.60728 | 0.20876 | 15.92592 | 0.20385 |
| 14 | 8.09434 | 0.09028 | 8.8822 | 0.20876 |
| 15 | 16.07052 | 0.16307 | 17.04869 | 0.09028 |
| 16 | 10.1132 | 0.08998 | 9.71708 | 0.18259 |
| 17 | 22.72339 | 0.43766 | 22.75584 | 0.46452 |
| 18 | 15.70144 | 0.18259 | 14.56596 | 0.06573 |
| 19 | 22.73741 | 0.46452 | 22.86641 | 0.03467 |
| 20 | 18.21783 | 0.07898 | 18.02566 | 0.10878 |

**S5D Table. ROE data from spinach plants inoculated with 10 nm TiO2-Ag NP at a concentration of 4%.**

| Monitoring days | Negative ROE behavior | Error | Positive ROE behavior | Error |
| --- | --- | --- | --- | --- |
| 1 | 16.95008 | 0.36073 | 16.9102 | 0.12703 |
| 2 | 7.61183 | 0.10551 | 7.61925 | 0.04742 |
| 3 | 9.47001 | 0.14642 | 9.09935 | 0.09295 |
| 4 | 14.21569 | 0.06722 | 14.1894 | 0.06274 |
| 5 | 12.52254 | 0.05152 | 12.52504 | 0.03772 |
| 6 | 19.57092 | 0.0829 | 19.56426 | 0.02855 |
| 7 | 6.90471 | 0.44634 | 6.98169 | 0.10574 |
| 8 | 20.75296 | 0.30611 | 21.00586 | 0.124 |
| 9 | 13.31647 | 0.18423 | 13.19603 | 0.21446 |
| 10 | 13.81369 | 0.28679 | 13.79601 | 0.1268 |
| 11 | 10.65468 | 0.07866 | 10.3129 | 0.18052 |
| 12 | 7.75501 | 0.15169 | 7.81472 | 0.29921 |
| 13 | 14.50348 | 0.25673 | 14.14134 | 0.16963 |
| 14 | 9.39877 | 0.28682 | 9.52443 | 0.10755 |
| 15 | 20.34279 | 0.16449 | 20.41299 | 0.29592 |
| 16 | 8.57402 | 0.29341 | 8.66399 | 0.10557 |
| 17 | 10.38191 | 0.07091 | 10.22629 | 0.1649 |
| 18 | 15.51746 | 0.20877 | 15.65111 | 0.77134 |
| 19 | 9.00685 | 0.22509 | 8.88869 | 0.16029 |
| 20 | 6.93537 | 0.22509 | 6.96315 | 0.01934 |

**S5E Table. ROE data from spinach plants inoculated with 10 nm TiO2-Ag NP at a concentration of 6%.**

| Monitoring days | Negative ROE behavior | Error | Positive ROE behavior | Error |
| --- | --- | --- | --- | --- |
| 1 | 8.81994 | 0.08522 | 8.7773 | 0.13648 |
| 2 | 10.55318 | 0.07551 | 10.65253 | 0.51276 |
| 3 | 7.79194 | 0.11965 | 7.82542 | 0.16923 |
| 4 | 16.33091 | 0.0644 | 16.19088 | 0.09124 |
| 5 | 11.71231 | 0.01406 | 11.86281 | 0.02667 |
| 6 | 8.81994 | 0.03063 | 8.7773 | 0.01935 |
| 7 | 10.55318 | 0.16625 | 10.65253 | 0.32184 |
| 8 | 7.79194 | 0.2954 | 7.82542 | 0.20917 |
| 9 | 16.33091 | 0.34259 | 16.19088 | 0.16779 |
| 10 | 17.15947 | 0.11965 | 17.21421 | 0.16923 |
| 11 | 12.053 | 0.34663 | 12.08368 | 0.38261 |
| 12 | 10.09344 | 0.08522 | 11.0006 | 0.13648 |
| 13 | 11.08398 | 0.07551 | 12.00256 | 0.51276 |
| 14 | 11.57638 | 0.11965 | 11.89811 | 0.16923 |
| 15 | 7.42626 | 0.0644 | 7.1405 | 0.09124 |
| 16 | 11.74798 | 0.01406 | 12.24422 | 0.02667 |
| 17 | 11.5392 | 0.03063 | 11.86313 | 0.01935 |
| 18 | 11.2448 | 0.16625 | 11.57122 | 0.32184 |
| 19 | 13.00793 | 0.2954 | 13.61313 | 0.20917 |
| 20 | 7.45089 | 0.34259 | 8.37287 | 0.16779 |
