# Supplementary material for: Effect of the suspension of Ag-incorporated TiO2 nanoparticles (Ag-TiO2 NPs) on certain growth, physiology and phytotoxicity parameters in spinach seedlings
Source: PLoS One. 2020 Dec 29;15(12):e0244511. doi: 10.1371/journal.pone.0244511 (PMC7771677; doi:10.1371/journal.pone.0244511)
Supplement: S6 Table — (DOCX) [file pone.0244511.s006.docx]

**S6 Table. ROE data from spinach plants inoculated with 26nm TiO_2_-Ag NPs at different concentrations (S6A 0%, S6B 0.25%, S6C 2%, S6D 4% and S6E 6%).**

**S6A Table. ROE data from spinach plants inoculated with 26 nm TiO_2_-Ag NP 0% concentration.**

| Monitoring days | Negative ROE behavior | Error | Positive ROE behavior | Error |
| --- | --- | --- | --- | --- |
| 1 | 19.61274 | 0.10611 | 19.50663 | 0.13147 |
| 2 | 21.92079 | 0.09424 | 21.23011 | 0.13371 |
| 3 | 21.41876 | 0.16609 | 21.49063 | 0 |
| 4 | 21.55031 | 0.12613 | 21.48577 | 0.01163 |
| 5 | 19.35605 | 0.15002 | 19.58909 | 0.03678 |
| 6 | 19.42259 | 0.15444 | 19.43784 | 0.11114 |
| 7 | 20.24185 | 0.05934 | 20.1859 | 0.12401 |
| 8 | 22.2973 | 0.09153 | 22.02478 | 0.14198 |
| 9 | 21.55031 | 0.01911 | 21.48577 | 0.01163 |
| 10 | 18.15837 | 0.41907 | 18.31637 | 0.05624 |
| 11 | 20.79946 | 0.0252 | 20.69199 | 0.40348 |
| 12 | 19.43348 | 0.4287 | 19.43348 | 0.05235 |
| 13 | 19.6752 | 0.49635 | 19.61885 | 0 |
| 14 | 22.1575 | 0.43054 | 21.8845 | 0.14195 |
| 15 | 18.84017 | 0.15615 | 18.84017 | 0.15356 |
| 16 | 18.28627 | 0.70173 | 18.30624 | 0.06738 |
| 17 | 19.97395 | 0.06069 | 19.66894 | 0.29307 |
| 18 | 18.94019 | 0.08278 | 18.63124 | 0.16307 |
| 19 | 18.72106 | 0.43949 | 18.87797 | 0.08998 |
| 20 | 22.58356 | 0.21981 | 22.47852 | 0.43766 |

**S6B Table. ROE data from spinach plants inoculated with 26 nm TiO_2_-Ag NP 0.25% concentration.**

| Monitoring days | Negative ROE behavior | Error | Positive ROE behavior | Error |
| --- | --- | --- | --- | --- |
| 1 | 15.15733 | 0.26325 | 15.15733 | 0.69488 |
| 2 | 8.4541 | 0.1223 | 8.54568 | 0.11767 |
| 3 | 8.85415 | 0.13659 | 8.73825 | 0.12201 |
| 4 | 14.85919 | 0.08694 | 14.8031 | 0.01365 |
| 5 | 17.39092 | 0.13659 | 17.63266 | 0.12201 |
| 6 | 18.86512 | 0.1223 | 18.86512 | 0.11767 |
| 7 | 11.62571 | 0.08248 | 11.73791 | 0.14013 |
| 8 | 6.55995 | 0.20412 | 7.78491 | 0.30944 |
| 9 | 7.63362 | 0.08248 | 8.17729 | 0.15761 |
| 10 | 11.48673 | 0.13942 | 11.99695 | 0.05825 |
| 11 | 17.72247 | 0.0584 | 17.83534 | 0.2219 |
| 12 | 15.99564 | 0.27735 | 16.13851 | 0.47083 |
| 13 | 16.96988 | 0.16628 | 17.176 | 0.05825 |
| 14 | 7.43446 | 0.0584 | 7.60558 | 0.47083 |
| 15 | 14.94602 | 0.16628 | 14.80019 | 0.16404 |
| 16 | 10.82783 | 0.31129 | 10.83468 | 0.31258 |
| 17 | 9.7863 | 0.31242 | 9.57367 | 0.35143 |
| 18 | 11.67367 | 0.48646 | 11.78714 | 0.16404 |
| 19 | 12.26999 | 0.35113 | 11.92886 | 0.35143 |
| 20 | 12.31543 | 0.31129 | 11.98891 | 0.27935 |

**S6C Table. ROE data from spinach plants inoculated with 26 nm TiO2-Ag NP at a concentration of 2%.**

| Monitoring days | Negative ROE behavior | Error | Positive ROE behavior | Error |
| --- | --- | --- | --- | --- |
| 1 | 12.93578 | 0.75528 | 13.33333 | 0.4856 |
| 2 | 15.75902 | 0.93055 | 15.12759 | 0.12037 |
| 3 | 8.10911 | 0.18425 | 8.16274 | 0.12915 |
| 4 | 10.12711 | 0.62336 | 9.7938 | 0.24504 |
| 5 | 7.73936 | 0.93055 | 8.27749 | 0.12037 |
| 6 | 6.05044 | 0.45919 | 6.05044 | 0.35047 |
| 7 | 11.23528 | 0.56012 | 10.09034 | 0.56915 |
| 8 | 9.52481 | 0.01905 | 9.41062 | 0.03496 |
| 9 | 12.21434 | 0.05181 | 13.90085 | 0.06468 |
| 10 | 10.57786 | 0.05198 | 10.33783 | 0.05198 |
| 11 | 13.78133 | 0.04095 | 12.32441 | 0.05054 |
| 12 | 8.91058 | 0.1682 | 8.92928 | 0.54211 |
| 13 | 11.71616 | 0.64116 | 10.4494 | 0.89269 |
| 14 | 12.02466 | 0.0892 | 12.02466 | 0.89269 |
| 15 | 4.83677 | 0.61559 | 4.26407 | 0.17657 |
| 16 | 12.4141 | 0.44648 | 10.52953 | 0.89269 |
| 17 | 8.15624 | 0.09607 | 8.84345 | 0.76066 |
| 18 | 10.71364 | 0.06689 | 11.59182 | 0.60997 |
| 19 | 8.05103 | 0.20315 | 7.56461 | 0.49085 |
| 20 | 10.39188 | 0.19949 | 10.64705 | 0.12077 |

**S6D Table. ROE data from spinach plants inoculated with 26 nm TiO2-Ag NP at a concentration of 4%.**

| Monitoring days | Negative ROE behavior | Error | Positive ROE behavior | Error |
| --- | --- | --- | --- | --- |
| 1 | 15.79467 | 0.2716 | 15.28694 | 0.26804 |
| 2 | 14.54355 | 0.09444 | 13.52729 | 0.10001 |
| 3 | 13.8936 | 0.17328 | 12.40645 | 0.15254 |
| 4 | 14.66496 | 0.17977 | 14.00572 | 0.17353 |
| 5 | 6.69391 | 0.05066 | 7.67088 | 0.02847 |
| 6 | 9.69587 | 0.20519 | 9.89543 | 0.13061 |
| 7 | 6.70316 | 0.07482 | 6.70316 | 0.11435 |
| 8 | 6.70316 | 0.11022 | 6.66117 | 0.09114 |
| 9 | 9.6979 | 0.25162 | 8.94473 | 0.09697 |
| 10 | 7.5358 | 0.0908 | 8.75087 | 0.10689 |
| 11 | 7.5358 | 0.13746 | 8.75087 | 0.0902 |
| 12 | 13.71714 | 0.15996 | 14.3134 | 0.09802 |
| 13 | 13.11048 | 0.23594 | 13.86829 | 0.27645 |
| 14 | 8.1584 | 0.51823 | 8.18758 | 0.50276 |
| 15 | 9.04323 | 0.22696 | 9.1492 | 0.32675 |
| 16 | 8.83593 | 0.32704 | 8.95485 | 0.36865 |
| 17 | 6.76982 | 0.08775 | 8.14971 | 0.10293 |
| 18 | 8.26023 | 0.06534 | 8.26023 | 0.04143 |
| 19 | 7.40439 | 0.20516 | 7.78276 | 0.48056 |
| 20 | 9.76439 | 0.11942 | 9.76439 | 0.20831 |

**S6E Table. ROE data from spinach plants inoculated with 26 nm TiO2-Ag NP at a concentration of 6%.**

| Monitoring days | Negative ROE behavior | Error | Positive ROE behavior | Error |
| --- | --- | --- | --- | --- |
| 1 | 9.01549 | 0.74408 | 8.49861 | 0.43846 |
| 2 | 6.55573 | 0.02253 | 5.78812 | 0.04612 |
| 3 | 6.29117 | 0.24803 | 6.8846 | 0.43846 |
| 4 | 15.90413 | 0.74408 | 15.51796 | 0.20137 |
| 5 | 5.37341 | 0.24048 | 5.53188 | 0.20137 |
| 6 | 11.92525 | 0.24048 | 11.92525 | 0.04873 |
| 7 | 7.03051 | 0.04778 | 7.73176 | 0.04859 |
| 8 | 5.70195 | 0 | 5.56212 | 0.09956 |
| 9 | 12.25694 | 0.09609 | 12.25694 | 0.70727 |
| 10 | 8.11731 | 0.68305 | 7.93762 | 0.129 |
| 11 | 7.81497 | 0.46604 | 7.19227 | 0.29434 |
| 12 | 9.54165 | 0.38303 | 9.54165 | 0.08329 |
| 13 | 8.05901 | 0.07007 | 8.84276 | 0.52892 |
| 14 | 7.95836 | 0.57744 | 7.95836 | 0.36977 |
| 15 | 7.55522 | 0.07007 | 7.21032 | 0.08329 |
| 16 | 11.41007 | 0.96798 | 11.41007 | 0.92804 |
| 17 | 9.10212 | 0.5102 | 9.10212 | 0.54657 |
| 18 | 11.37212 | 0.07007 | 11.37212 | 0.08329 |
| 19 | 14.94744 | 0.10025 | 14.94744 | 0.10782 |
| 20 | 7.01055 | 0.07007 | 7.01055 | 0.92804 |
